# New insights into the structure and dynamics of the epigenetic modifications on DNA

**DOI:** 10.1039/d5cb00207a

**Published:** 2025-10-13

**Authors:** Dineshbabu Takkella, Javier Cerezo, Lara Martinez-Fernandez, Krishna Gavvala

**Affiliations:** a Department of Chemistry, Indian Institute of Technology Hyderabad Kandi Sangareddy Telangana-502284 India kgavvala@chy.iith.ac.in; b Departamento de Química, Facultad de Ciencias and Institute for Advanced Research in Chemistry (IADCHEM), Universidad Autónoma de Madrid Campus de Excelencia UAM-CSIC Cantoblanco, 28049 Madrid Spain; c Departamento de Química Física de Materiales, Instituto de Química Física Blas Cabrera, Consejo Superior de Investigaciones Científicas, IQF-CSIC Calle Serrano 119 28006 Madrid Spain lmartinez@iqf.csic.es

## Abstract

DNA methylation is a key epigenetic modification involved in genomic imprinting, X-chromosome inactivation, and repression of repetitive element transcription and transposition. Despite its biological significance, the impact of epigenetic modifications such as methylcytosine (mC) and hydroxymethylcytosine (hmC) on the structural and UV-induced dynamics of DNA remains poorly understood. Here, we employed the fluorescent nucleobase analogue 2-aminopurine (2Ap) in combination with steady-state and time-resolved spectroscopy, molecular dynamics, and quantum mechanical calculations to investigate these effects. Our findings reveal distinct differences in base stacking and helical stability between mC and hmC-modified DNA. mC-modified DNA predominantly adopts a stacked conformation, promoting efficient fluorescence quenching of 2Ap. In contrast, hmC-modified DNA displays both stacked and non-stacked conformations, leading to reduced base stacking and a more hydrophobic local environment, as indicated by blue-shifted emission spectra. Furthermore, although charge-transfer quenching occurs in all systems, hmC shows weaker charge-transfer character, resulting in higher fluorescence quantum yields and longer lifetimes. These results highlight the subtle but crucial role of hmC in modulating local DNA conformation and stability. Moreover, they demonstrate the effectiveness of 2Ap as a sensitive probe for detecting epigenetic modifications, offering deeper insights into the molecular mechanisms of DNA methylation and demethylation pathways.

## Abstract 2

2-Aminopurine fluorescence and simulations show that epigenetic modifications tune DNA structure and dynamics, with methylcytosine stabilizing stacking and hydroxymethylcytosine inducing flexible, less stacked conformations.

## Graphical abstract

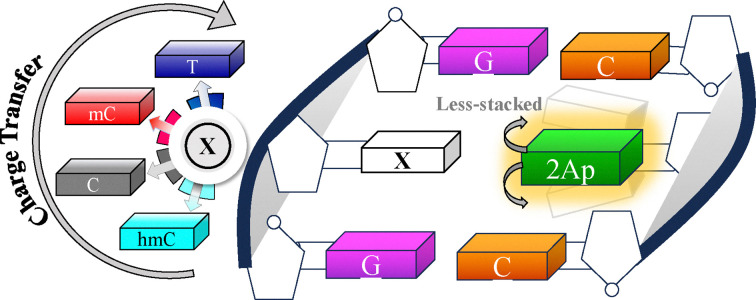

## 1. Introduction

**Scheme 1:**
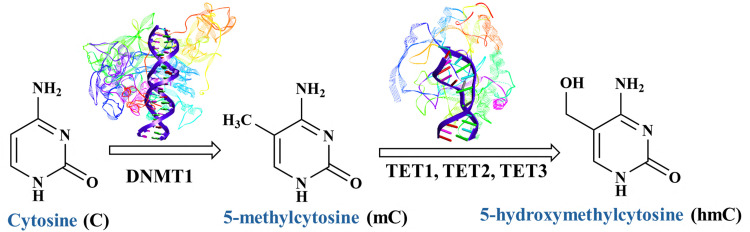
Chemical structures of cytosine and its epigenetic modifications on DNA induced by DNMT1 (PDB ID: 3PTA) and TET (PDB ID: 7NE3) enzymes.

Genome is the major structural component of the cell that provides all the genetic information required for the growth and function of an organism. Modifications on the genome without altering the sequence are usually known as epigenetic modifications^1,2^ and are essential for regulating cellular functions.^1–3^ Therefore, the cell fate relies largely on the epigenetic Scheme 1. Chemical structures of cytosine and its epigenetic modifications on DNA induced by DNMT1 (PDB ID: 3PTA) and TET (PDB ID: 7NE3) enzymes mechanisms that control gene expression, which is critical for various biological and pathological processes.^1,2^ DNA methylation, one of the key epigenetic modifications, involves the transfer of a methyl group from the cofactor *S*-adenosyl-l-methionine (SAM) to specific sites in the DNA.^4–6^ In eukaryotic cells, methylation takes place specifically on the C5 position of cytosine, predominantly within CpG islands of DNA.^7–9^ Methylated cytosine (mC)^10,11^ is often referred to as the fifth base of human DNA, accounting for roughly 1% of the bases in mammalian genomes,^12^ and has a role in a plethora of biological processes, either functional or pathological.^6,13–17^ This heritable DNA modification on cytosine base is catalysed by enzymes known as DNA methyltransferases (DNMTs). Subsequently, TET (ten-eleven-translocation) enzymes oxidizes 5-methylcytosine (mC) to 5-hydroxymethylcytosine (hmC) and further to 5-formyl cytosine (fC) and 5-carboxyl cytosine (caC) as shown in Scheme 1.^4,5^ Though the oxidation mechanism of mC is well established, and their epigenetic role has been studied,^18–25^ there is still room to understand their physical and conformational effects on DNA flexibility and its ability to wrap around histones to form nucleosomes.^26^ Beyond this structural impact on DNA, the presence of these epigenetic bases, especially mC, has been connected with increase UV-induced damage,^13,14,17,27–29^ pointing also to effects into DNA photoinduced dynamics. To fully unravel these sequence-dependent effects within the nearest-neighbour framework, we explored these issues using a variety of experimental and theoretical approaches.

**Fig. 1:**
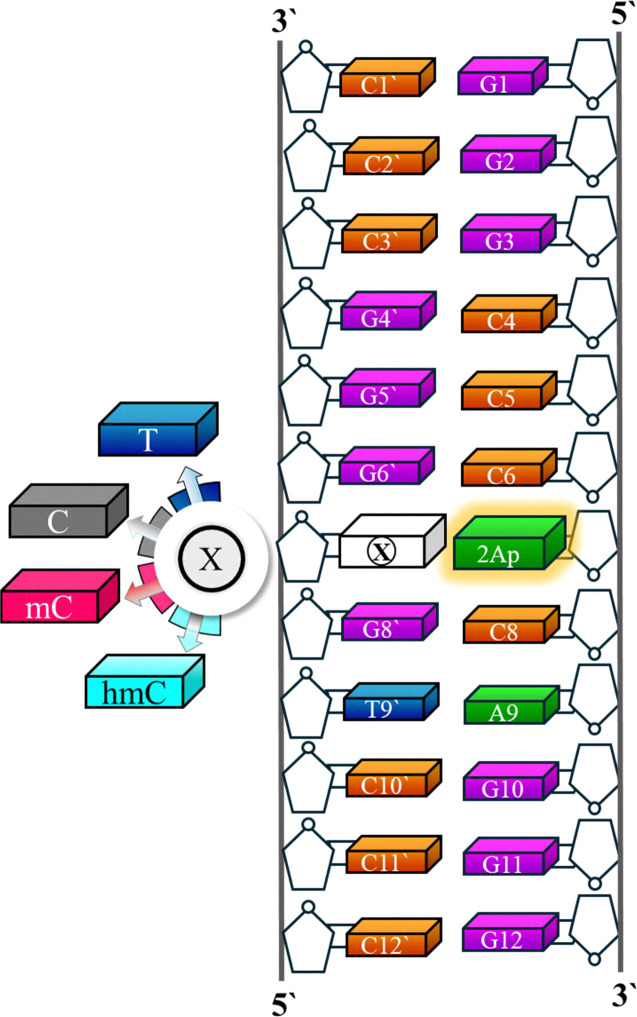
Schematic representation of sequences of the 2Ap-labeled DNA duplexes and base labeling used in this study.

In detail, we first examined the structure (using thermal melting, circular dichroism, and molecular dynamic (MD) simulations) and dynamics of 12 bp (base pairs) duplexes containing epigenetic bases and labeled with 2-aminopurine (2Ap). As a model DNA duplex, we use a 12-bp duplex in which the A7 residue is replaced by 2Ap (Fig. 1). The ssDNA sequence is 5′-GGGCCC**2Ap**CAGGG-3′ (C**2Ap**C). In the corresponding dsDNA duplexes, the WC base pair of 2Ap is either C, T, mC, or hmC, denoted as C2ApC/GCG, C2ApC/GTG, C2ApC/GmCG, and C2ApC/GhmCG, respectively. In native CpG islands, cytosine (including mC and hmC) pairs with guanine; however, in this work, we deliberately positioned 2Ap opposite the modified cytosine. 2Ap's well-established ability to form wobble-like yet stable base pairs with cytosine.^30^ Although structurally distinct from guanine, 2Ap preserves local duplex stability and exhibits high sensitivity to microenvironmental changes.^31–33^ Embedding 2Ap within a GC-rich sequence that retains CpG-like structural features allowed us to probe the local structural and photophysical effects of mC/hmC modifications in a physiologically relevant context. Then, to enhance the understanding of the photophysics of 2Ap-labeled DNA duplexes and their dependence on DNA sequence context, we investigate the UV absorption, emission, and time-resolved fluorescence, systematically varying the composition of bases opposite 2Ap. 2Ap has been selected as a fluorescent analogue since it can be incorporated into DNA, replacing the natural base without significantly disrupting DNA structure or its interactions with proteins.^34–36^ Furthermore, 2Ap is highly responsive to its microenvironment, especially to stacking interactions, offering insights into its local environment,^31–33^ which is of particular importance in our study. In this setup, we maintained a constant composition of the neighbouring bases (cytosine) and varied the opposite bases of 2Ap. The experimental data were complemented with MD simulations, which provide atomistic insight into the structural effects induced by 2Ap, and quantum mechanical (QM) calculations to elucidate the key photophysical processes (excited state nature and decays) in each duplex. Our integrated analysis offers an explanation for the observed spectroscopic properties and their dependence on 2Ap's neighbouring or opposite nucleobases, clarifying the various factors that influence the photophysical characteristics of 2Ap. These dynamic interaction properties could offer detailed information about the effectiveness and continuity of epigenetic replication and would be valuable for gaining further insights into conformational changes or protein binding events by analysing observed photophysical changes in relation to structural and electronic effects. Altogether, these discoveries uncovered a previously elusive mechanism of active DNA demethylation, ultimately leading to a wave of research in mammalian epigenetic regulation.

## 2. Materials and methods

### 2.1. DNA Sequences

All labeled and non-labeled DNA sequences were purchased from Integrated DNA Technologies, Inc. (U.S.A.). The 12-bp duplex oligonucleotide (ODN) sequence used here was 5′-GGGCCC**2Ap**CAGGG-3′/5′-CCCTG**X**GGGCCC3′ where X represents a modification site. In this study, the sequence has either matched (X = thymine, T) or mismatched base pairs (X= C, mC, and hmC) at the X position. The hemimethylated modifications included the incorporation of cytosine (C), methyl cytosine (mC), or hydroxymethyl cytosine (hmC) at the modification site. 2Ap-labeled dsDNA duplexes were prepared by heating 30 μM of 2Ap-labeled ssDNA sequence and 30 μM complementary matched or mismatched ssDNA sequences at 90 °C for 5 minutes, followed by slow cooling to room temperature. All experiments were conducted in a buffer (pH 7.5) consisting of 25 mM TRIS-HCl with 150 mM NaCl.

### 2.2. Absorption spectroscopy

UV-Vis absorption spectra were recorded on the JASCO V-730 spectrophotometer. The concentration of single-stranded sequences was governed using extinction coefficients at 260 nm. The extinction coefficients for the 2Ap-labeled sequence 5′-GGGCCC**2Ap**CAGGG-3′ was 104 000 cm^−1^ M^−1^. Extinction coefficients for the nonlabeled single stranded sequences 5′-CCCTG**T**GGGCCC3′, 5′-CCCTG**C**GGGCCC3′, 5′-CCCTG**mC**GGGCCC3′ and 5′-CCCTG**hmC**GGGCCC3′ were 99 400 cm^−1^ M^−1^, 97 300 cm^−1^ M^−1^, 95 600 cm^−1^ M^−1^, and 97 300 cm^−1^ M^−1^, respectively.

### 2.3. Fluorescence spectroscopy

The steady-state fluorescence spectra were recorded on the JASCO FP-8350 spectrofluorometer. For steady-state fluorescence studies, matched and mismatched 2Ap-labeled duplexes were excited at wavelengths of 290 nm and 315 nm. The excitation wavelength of 315 nm was selected to specifically excite the 2Ap. The measurements were supervised on 5 μM ODN duplexes. The apparent relative quantum yields (QYs) of sequence-dependent DNA in Tris-HCl buffer were determined using alone 2Ap as a standard (*Φ* = 0.68).^37^ Relative QYs were calculated from eqn (1).1
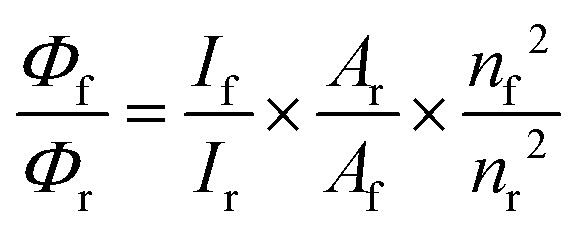
The subscripts f and r denote the sample and reference molecules, respectively. Here, *Φ* denotes the quantum yield, *I* represents the integrated area under the fluorescence emission spectrum, *A* denotes the absorbance at 315 nm, and *n* values correspond to the refractive index of the solvents used.

The melting curves of the duplexes (2 μM) in 25 mM TRIS-HCl buffer (pH = 7.5) with 150 mM NaCl were measured by monitoring the temperature-dependent fluorescence changes at 370 nm using a Horiba Fluoromax-4 spectrofluorometer equipped with a Peltier system controlled by a Thermo Scientific NESLAB RTE-7. The temperature was escalated from 15 to 95 °C at a heating rate of 0.5 °C min^−1^. Melting temperatures were obtained from the melting curves following the method outlined in previous literature.^38^

### 2.4. Time-resolved fluorescence spectroscopy

Fluorescence lifetime was recorded using TCSPC (time-correlated single photon counting) setup by Horiba (FluoroHub). Fluorescence decays were measured at 291 nm (NanoLED) excitation wavelength with an IRF (Instrumental response function) of <1 ns. Fluorescence decays were processed using Horiba's DAS6 package by convolving the IRF with exponential decay functions. The fitting was performed following the mathematical model defined in eqn (2).^39^2
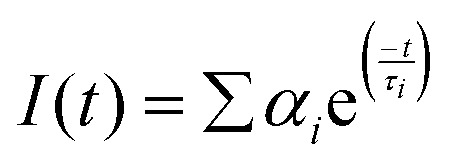
where *I*(*t*) signifies the exponential fluorescence decay, *τ*_*i*_ denotes fluorescence lifetime, and *α*_*i*_ represents the fractional contribution of the *i*th component. In TCSPC experiments, the average error was ≤5%.

A global analysis method was executed to analyze multi-wavelength decay data, enhancing the accuracy of decay component determination.^40,41^ This approach assumes that the decay components *τ*_*i*_ are independent of the emission wavelength, while their amplitudes *α*_*i*_ change with wavelength. The DAS6 analysis tool incorporates an integrated global analysis fitting module. The DAS (Decay-associated spectra) were built based on eqn (3).3
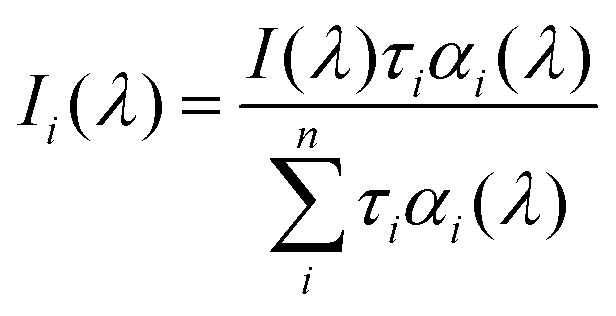
Here, *I*(*λ*) represents the steady-state emission spectrum, while *α*_*i*_(*λ*) denotes the wavelength-dependent amplitude. The DAS offers the spectra for each lifetime component, thereby revealing the intensity contribution of individual species to the total emission of the duplex. The real amplitudes corresponding to each lifetime species were calculated from 
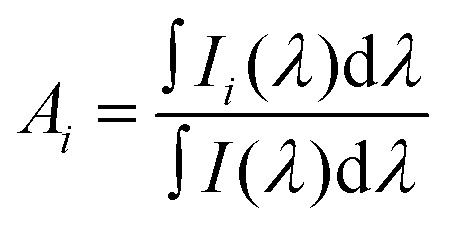
, here the numerator denotes the area under the DAS associated with species *i*, while the denominator represents the area under the steady-state emission spectrum.

### 2.5. Circular dichroism spectroscopy

The CD (circular dichroism) spectra were measured using a JASCO J-815 spectrophotometer. Each CD spectrum was the result of averaging three accumulated spectra of the sample and was recorded at a scanning speed of 100 nm min^−1^. Each measurement's baseline was recorded using a 25 mM Tris-HCl buffer solution with 150 mM NaCl, at pH 7.5. The concentration of each dsDNA sequence used in the experiment was measured to be 5 μM.

### 2.6. Molecular dynamics simulations

A DNA double-strand with standard bases (GGGCCCGCAGGG) was generated using the DNA/RNA builder tool implemented in Avogadro.^42^ The structure was subsequently modified with the same molecular editor to generate the non-standard versions, including 2Ap, mC, and hmC bases (Scheme S1). Interactions were treated with the AMBER-consistent parmbsc1 force field (FF),^43^ which includes all standard nucleotides, as well as the solvent and counterions (Na^+^). FF parameters for non-standard nucleotides (2Ap, mC, and hmC) were derived by selecting atom types within the FF by analogy and adding any missing bonded terms by similarity with those already implemented in the FF. Restricted electrostatic potential fitted (RESP) charges for non-standard residues were obtained using antechamber^44^ at the wB97XD^45^/cc-pVDZ level of theory, using Gaussian16.^46^ The charges were adjusted to retain those of the sugar-phosphate backbone from standard AMBER while updating those in the nucleotide region, ensuring proper charge balance. This protocol was selected since its application to standard residues yields charges very similar to those included in the AMBER FF, validating the consistency of the derived parameters. The full parameter set is provided in Tables S1 and S2 of the SI. MD simulations were carried out using GROMACS 2021.4.^47^ The DNA strands generated with Avogadro were solvated in a dodecahedral box, ensuring at least a 20 Å buffer between the strand and any box edge, followed by neutralization of the negative charges with sodium cations. The system was, then, energy-minimized to remove steric clashes and equilibrated under an NVT ensemble at 298.15 K for 500 ps using the Nosé–Hoover thermostat (*s* = 1 ps).^48,49^ The production run was performed under an NPT ensemble at 298.15 K and 1 atm using the Nosé–Hoover thermostat (*τ* = 1 ps) and the Parrinello–Rahman barostat (*τ* = 5 ps)^50^ for a total of 100 ns. Comparison with shorter trajectories (10 ns, shown in Fig. S1), confirm that the 100 ns trajectories are converged with respect to the structural parameters analysed. In all simulations, electrostatic interactions were evaluated using the PME algorithm,^51^ with a real-part cutoff of 11 Å, while vdW interactions were treated with an 11 Å cutoff. A time step of 1 fs was employed, with constraints applied to all bonds involving hydrogen atoms with LINCS.^52^ Additional simulations with a larger timestep of 2 fs (Fig. S1) provided similar results for one of the systems (T), and could be considered for follow-up investigations. The simulations were analysed by measuring the stacking angle between 2Ap and the adjacent bases within the same strand. This was quantified as the angle between the normal vectors to each ring plane, determined *via* the cross-product of vectors defined by selected atoms in each ring. The distribution of the stacking angles over the trajectories are obtained with an histogram with a bandwidth of 1°. The evolution of the stacking angle over the trajectories is shown in Fig. S2.

To assess statistical uncertainty in the stacking-angle distributions (Fig. S1 and S3), we applied a moving-block bootstrap.^53^ For each trajectory, the time series of stacking angles was first analysed through the autocorrelation function to obtain the integrated autocorrelation time, *τ*_int_, which ranged from ∼10 ps up to several hundred ps depending on the system and analysed base. Block lengths of 5*τ*_int_ were then used to resample the time series while preserving time correlations. For each system, 1000 bootstrap replicas were generated and histograms recomputed. The distributions were then plotted including the resulting 95% confidence bands for the probability density in each bin.

### 2.7. Quantum mechanical calculations

From the extracted MD geometries, selected as representative from the lower and upper extremes of the stacking-angle distribution, one for each system, we have then optimized their ground state minima (*S*_0min_) using density functional theory (DFT) and in particular the M052X^54,55^ functional combined with the 6-31G(d) basis set. This method has been selected due to its good performance when treating this kind of system prone to populate charge transfer states^54^ and has also already been tested for other fluorescent derivatives inside DNA.^56^ Our QM model is based on the hexamer formed by C62ApC8/G6’XG8’ (Fig. 1). Solvent (water) effects were also considered through an implicit polarizable continuum model (PCM).^57,58^ Then, the absorption energies, intensities, and excited state character were obtained at this *S*_0min_ resorting to the time-dependent version of DFT (TD-DFT). Subsequently, the excited state potential energy surface was mapped by relaxation of the lowest-lying states at the same level of theory. At this point, the equilibrium version of PCM was selected as the most appropriate. The amount of charge transfer is estimated just through the difference between the Mulliken charges in the ground and excited state. All the calculations were done with Gaussian16 program.^46^

## 3. Results

### 3.1. Conformational/structural effect of DNA methylation markers

#### 3.1.1. Thermal melting and circular dichroism analysis of epigenetic DNA duplexes

**Fig. 2:**
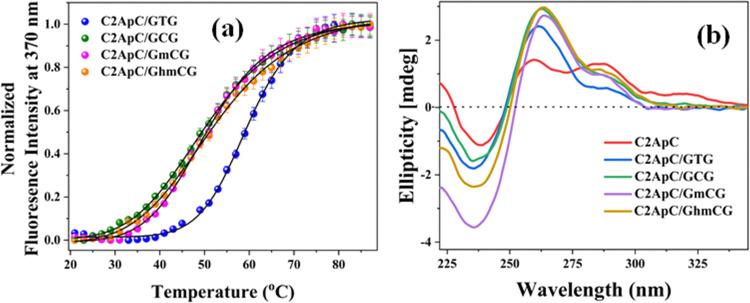
Melting temperature curves (a) and CD spectra (b) of the 2Ap-labeled ssDNA matched, mismatched modified dsDNA sequences.

The stability of duplexes was measured by their melting temperature (*T*_m_), which was experimentally determined for the 2Ap-labeled DNA duplexes through temperature-dependent changes in fluorescence emission intensity at 370 nm (Fig. 2a and Table S3). Interestingly, the experimental melting temperature (*T*_m_ = 59.5 ± 0.14 °C) for the C2ApC/GTG duplex closely matched the *T*_m_ predicted by DINAMelt^59^ (58.5 °C) for the 5′-GGGCCC**A**CAGGG-3′/5′-CCCTG**T**GGGCCC-3′. This observation confirms that replacing adenine with 2Ap does not remarkably alter the melting temperature of DNA duplexes. As expected from the higher stability of 2Ap-T base pairs compared to 2Ap-C base pairs, the C2ApC/GTG duplexes exhibited significant thermal stability, with a *T*_m_ increase of ∼9 ± 0.16 °C compared to C2ApC/GCG duplexes. The lower *T*_m_ values suggest that mismatched sequences are minimally stable compared to matched sequences. In the case of 2Ap-C base sequences, alterations from C to mC or hmC caused distinct changes in *T*_m_. The C2ApC/GmCG and C2ApC/GhmCG duplexes exhibited only a very slight *T*_m_ increase of ∼0.4 to 0.8 °C compared to C2ApC/GCG. The slight *T*_m_ increase may have originated from the marginal stabilization of mC and hmC compared to C. To further validate the effects of cytosine modifications, the *T*_m_ of duplexes containing a template guanine (G) paired with C, mC, and hmC on the complementary strand was measured (Fig. S4 and Table S4). The *T*_m_ of CGC/GCG (60.4 ± 0.4 °C) reflects higher stability than that of C2ApC/GCG (50.11 ± 0.17 °C). The CGC/GmCG reduces *T*_m_ by ∼2.5 °C, and CGC/GhmCG by ∼12 °C, compared to CGC/GCG, indicating that cytosine modifications destabilize G-based duplexes. Conversely, in 2Ap-C mismatches, mC and hmC slightly change *T*_m_, indicating weak stabilization. These results highlight that, although 2Ap does not fully mimic guanine in base pairing, it remains a reliable fluorescent probe for detecting subtle stability changes induced by cytosine modifications.

2Ap-DNA duplexes show positive CD bands (Fig. 2b) in the ∼260 and ∼290 nm along with a negative band near ∼245 nm, which are typical spectral features of the B-form DNA structure.^32,60^ The CD spectra of 2Ap-labeled ssDNA exhibit one extra positive peak at 325 nm, which corresponds to stacking interactions of adjacent 2Ap residues.^60^ The 260 nm peak relates to base stacking interactions, while the 245 nm peak is associated with the helical structure of the DNA. There was a significant increase in ellipticity for both positive and negative bands in 2Ap-dsDNA compared to 2Ap-labeled ssDNA duplex. The lower ellipticity of the positive band in 2Ap-T base pair dsDNA compared to 2Ap-C base pair dsDNA is consistent with similar results examined in previous literature.^61^ C2ApC/GCG, C2ApC/GmCG, and C2ApC/GhmCG duplexes exhibit equal ellipticity at the positive band, with a minor red shift in the peak position. C2ApC/GhmCG duplexes exhibited a ∼2 nm red shift in the positive band when associated with the C2ApC/GTG duplexes, revealing subtle differences in their structural properties. A similar trend occurred at the 290 nm positive peak, where the C2ApC/GTG duplex exhibited lower ellipticity than the C2ApC/GCG and C2ApC/GmCG duplexes. The C2ApC/GhmCG duplex shows a slight increase in ellipticity at 290 nm compared to C2ApC/GCG and C2ApC/GmCG, suggesting that hmC induces subtle structural changes, possibly affecting helical conformation and base stacking.

#### 3.1.2. Molecular dynamics

**Fig. 3:**
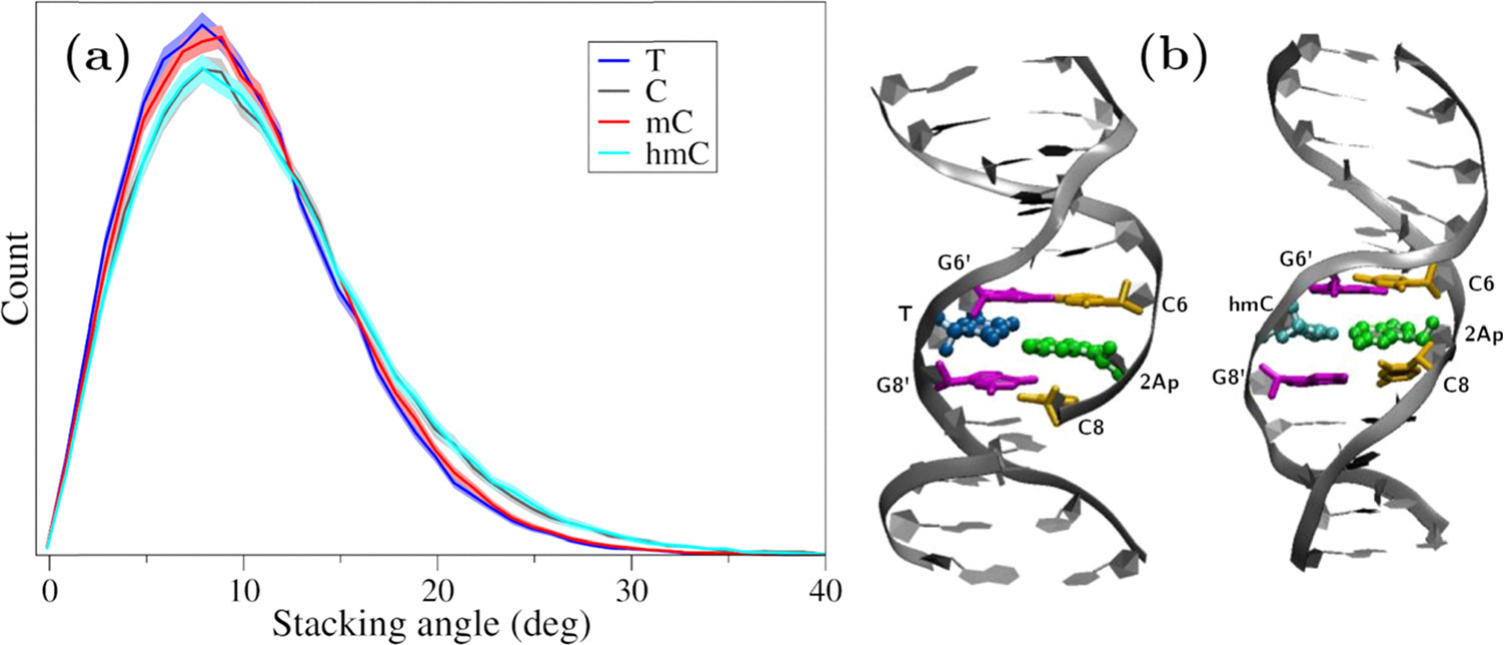
(a) Stacking-angle distributions between 2Ap and C8 for duplexes containing the different X bases. Average angles are 10.0° (T), 11.0° (C), 10.2° (mC), and 11.1° (hmC). Shaded regions denote 95% confidence intervals obtained from block-bootstrap analysis. (b) Representative structures for T and hmC, including the labels for the nearby bases (color code: C6/C8 in orange, 2Ap in green; G6/G8 in magenta; T in blue, and hmC in cyan).

In short, both thermal and CD experimental results point to moderate effects on the structure of 2Ap-labeled modified duplexes for the studied bases. To further assess this issue, classical MD simulations have been performed. After a 100 ns run, the stacking degree among adjacent bases was analyzed, being determined as the angle between the vectors perpendicular to each ring (Section 2.6), namely, the co-planarity of adjacent basis increases as the stacking angle approaches zero. The planarity of the consecutive 2Ap and C8 residues depends on X (recall Fig. 1 for labels), as shown in Fig. 3a, which reports the distribution of the corresponding stacking angle. The shaded regions denote the 95% confidence intervals obtained by block bootstrap; their narrow extent confirms that the observed differences and similarities between bases are statistically significant. Specifically, both hmC and C promote less-stacked configurations, with stacking angle distributions with broader tails toward non-planar geometries and reduced probability density around the maximum, whereas T and mC better preserve the co-planarity between 2Ap and C8. It should be noted that the tail of the distribution in Fig. 3a corresponds to transient spikes observed along the trajectory, which persist for only a few picoseconds. The more pronounced tails for hmC and C arise from more frequent distortions reaching larger stacking angles, as illustrated in Fig. S2. Despite this difference, completely solvent-exposed or unstacked conformations have not been located along the dynamics. Interestingly, the nature of the X base has a negligible effect on the stacking degree between 2Ap and C6, whereas the stacking of X with G6′ or G8′, which determines how the base accommodates within the strand, shows some dependence on the identity of X (see Fig. S3). Thus, although the global duplex structure is maintained, in agreement with the experimental results, the different nucleobases exhibit slightly different stacking patterns, with hmC showing the largest deviation from co-planarity.

To further confirm this hypothesis two “extreme” cases being X = T and hmC, were extracted from the MD (Fig. 3b) and reoptimized at the DFT level of theory, using as a model the hexamer formed by C6-2Ap-C8 + G6′-X-G8′. For each system representative cases of stacked (*i.e.*, configurations with stacking angles below 1 degree) and less-stacked (*i.e.*, configurations with stacking angles over 30 degrees) conformations were considered. Upon geometry optimization, it is confirmed that T favours stacked 2Ap-C8 arrangements (angles lower than 15 degrees), since even when starting from large angles, the optimization converged to smaller ones; whereas the opposite occurs for hmC, leading to less-stacked ones (angles between 25–35 degrees) even when starting from lower ones. Once it has been confirmed that, although small, some structural changes are induced by the modified bases (especially for hmC), their impact on the absorption/emission of UV light (Section 3.2) as well as in the excited state dynamics (Section 3.3) will be studied.

### 3.2. Effect on 2Ap-DNA photophysical properties

#### 3.2.1. Steady-state absorption and emission properties of 2Ap-labeled DNA

**Fig. 4:**
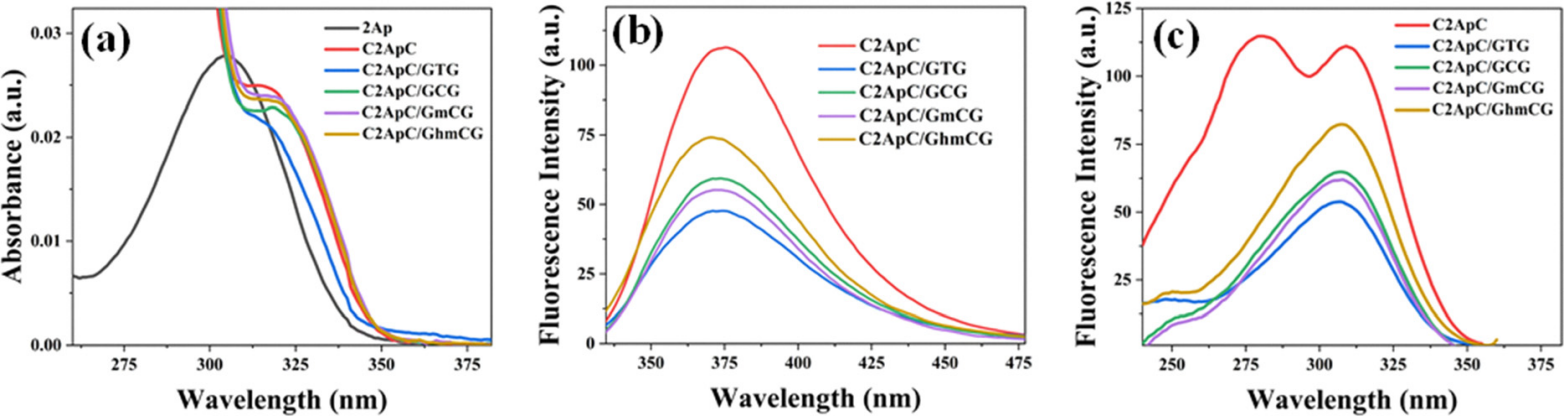
Absorption (a) and fluorescence emission ((b) collected at 315 nm excitation wavelength) and excitation ((c) collected at 370 nm emission wavelength) spectra of 2Ap, labeled ssDNA, matched, mismatched, methylated, and hydroxymethylated dsDNA sequences.

The UV absorption spectra for ssDNA, as well as matched and mismatched dsDNA duplexes, were measured (Fig. 4a). In the 2Ap-labeled DNA duplexes, UV absorption in the 240–280 nm range is governed by the normal bases. This dominance occurs from 2Ap's weak absorbance (0.2%) at 260 nm and its 11 : 1 ratio to normal bases.^62^ A small 2Ap absorption shoulder band appears at 307–315 nm, enabling accurate estimation of base contributions,^36,63^ since the main absorption band of 2Ap-labeled DNA is red-shifted relative to the absorption band of free 2Ap. Unlike ssDNA, dsDNA contains stacked bases that increase its stability and decrease its absorbance (hypochromicity).^64^Fig. 4a clearly illustrates a decrease in absorbance for dsDNA duplexes compared to ssDNA (C2ApC). The monitored hypochromic effect suggests that Watson–Crick (WC) pairing with a complementary C2ApC/GTG residue promotes optimal stacking of 2Ap with adjacent nucleobases, as already discussed in Section 3.1. In contrast, mismatched duplexes (C2ApC/GCG, C2ApC/GmCG, and C2ApC/GhmCG) show a small increase in absorbance compared to matched duplexes, which may be due to the structural deviation of the flanking base pairs. Nonetheless, all the duplex sequences exhibit same absorption maximum with slightly different amplitudes. The fluorescence emission spectra were measured upon excitation at 290 nm (Fig. S5) and 315 nm (Fig. 4b). As shown in Fig. 4b, ssDNA has a huge emission intensity compared to dsDNA duplexes. The complementary sequence of C2ApC/GTG duplex showed very low emission intensity compared to ssDNA. Mismatched DNA duplexes display higher fluorescence intensity compared to matched sequences. This observation could be related to reduced base-pair stability or conformational changes in the DNA duplexes, potentially induced by cytosine methylation status. Despite the intensity changes, spectral shifts were observed (Fig. S6a). A slight blue shift (∼6 nm) was monitored in the dsDNA duplex emission spectra compared to the ssDNA. The emission maxima were ∼376 nm, ∼374 nm, ∼374 nm, ∼372 nm, and ∼370 nm for C2ApC, C2ApC/GTG, C2ApC/GCG, C2ApC/GmCG, and C2ApC/GhmCG, respectively. C2ApC/GhmCG exhibits distinct spectral changes, displaying a 2 nm blue shift compared to C2ApC/GmCG and a 4 nm blue shift compared to C2ApC/GCG.

The fluorescence excitation spectrum of the 2Ap-labeled ssDNA exhibits two excitation bands (Fig. 4c). The initial band of the excitation spectrum lies between 245 and 285 nm, representing the energy transfer from adjacent DNA bases to 2Ap. The fluorescence intensity in the initial band region correlates with energy transfer efficiency, which increases with lower temperatures.^62^ A second excitation band represents the direct excitation peak of 2Ap (∼309 nm, which is consistent with the shoulder observed in the absorption spectrum, also located in this region of the spectrum and associated with 2Ap absorption). In the case of 2Ap-labeled dsDNA duplexes, a single excitation band is detected at ∼306 nm. At temperatures above 15 °C, it is not feasible to significantly visualize a second excitation band, typically between 255 and 280 nm.^65^ Furthermore, dsDNA duplexes show ∼50% lower energy transfer efficiency related to ssDNA, which is why the initial excitation band is not observable in dsDNA at room temperature.^62^ The slight blue shift (∼3 nm) in the excitation band of 2Ap-labeled dsDNA compared to ssDNA is attributed to enhanced base stacking interactions in the duplex (Fig. S6b). This shift reflects the structural rigidity and reduced solvent exposure of the 2Ap bases. The excitation spectra also reflected the trends observed in the emission intensity changes for matched and mismatched DNA duplexes.

The QYs of 2Ap-labeled DNA duplexes were significantly lower than those observed for free 2Ap.^66^ The QY of ssDNA (C2ApC) was 0.0173, while that of C2ApC/GTG was 0.0049 (Section 3.3). This decrease was attributed to significant stacking interactions between T and 2Ap in the dsDNA duplex. The relative QY of C2ApC/GTG is relatively close to those of C2ApC/GCG (0.0063) and C2ApC/GmCG (0.0059) (Section 3.3), although below them. In the case of C2ApC/GhmCG, the QY (0.01) is approximately double that of C2ApC/GCG and C2ApC/GTG.

#### 3.2.2. Vertical absorption and emission energies

The absorption spectra of the labeled duplexes were then simulated by computing the vertical absorption energies and intensities on top of the optimized ground state minima of the four systems (T-stacked and less-stacked and hmC-stacked and less stacked). The absorption maxima and the global shape of the spectra are almost unaffected either by the nature of the X base or by the stacking degree (Fig. S7), in agreement with the experimental trends (Fig. 4a). A more detailed analysis of the lowest lying excited states is collected in Table 1 and Table S5). For the four systems, the first two excited states are located in 2Ap, being the S_1_ bright and S_2_ dark. Then, the third state S_3_ is located on the X nucleobase, and it is dark for T and partially “bright” for hmC. The following states arise from mixtures between the bright ππ* states of G's and C's and excited state with different amounts of charge transfer (CT). Among the four studied geometries, hmC-stacked stands in terms of CT stability with the S_6_ showing a strong CT character involving 2Ap and C8 nucleobases (Fig. S8). Some conclusions can be extracted from the close inspection of Table 1 (Table S5): (1) stacked systems have slightly more CT compared to less-stacked systems, and (2) the stacking degree also affects the nature of the bases involved in the first clear CT state, being 2Ap and C8 in stacked ones and G8 and X in the less stacked systems.

Excited state character, vertical absorption energies (in eV), oscillator strength, and charge transfer character (in a.u.) computed at the PCM/TD-M052X/6-31G(d) level of theory

**Table:** 

	Character	Δ*E*	*f*	CT	Character	Δ*E*	*f*	CT
	T-less-stacked				T-stacked			
S_1_	ππ* 2aP	4.71	0.2187	0.0	ππ* 2aP	4.62	0.1870	0.0
S_2_	nπ* 2aP	5.07	0.0052	0.0	nπ* 2aP	5.06	0.0048	0.0
S_3_	nπ* T	5.17	0.0096	0.0	nπ* T	5.17	0.0070	0.0
S_4_	ππ* G6′	5.18	0.0689	0.1	ππ* G6′_+C6+C8_	5.21	0.0968	0.1
S_5_	ππ* G8′	5.21	0.0613	0.1	ππ* G8′_+C6+C8_	5.21	0.1018	0.1
S_6_	ππ* C6	5.29	0.1663	0.1	ππ* G8′ + CT	5.24	0.0881	0.3
S_7_	ππ* C8_+C6_	5.29	0.3268	0.1	ππ* C6_+G6′_	5.27	0.1788	0.1
S_8_	CT G8- > T	5.38	0.0538	0.7	CT 2aP- > C8	5.32	0.1576	0.5

**Table:** 

	Character	Δ*E*	*f*	CT	Character	Δ*E*	*f*	CT
	hmC-less-stacked				hmC-stacked			
S_1_	ππ* 2aP	4.59	0.2119	0.0	ππ* 2aP	4.50	0.1507	0.1
S_2_	nπ* 2aP	4.96	0.0035	0.0	nπ* 2aP	4.95	0.0078	0.0
S_3_	ππ* hmC	5.06	0.1152	0.0	ππ* hmC	5.06	0.0798	0.2
S_4_	ππ* G6′_+C6_	5.23	0.0995	0.1	ππ* G6′_+C6_	5.21	0.1498	0.1
S_5_	ππ* G8′_+C8_	5.26	0.0408	0.1	ππ* G8′_+C8_	5.25	0.0796	0.0
S_6_	ππ* C6 _+G6′_	5.30	0.1478	0.0	CT 2aP-> C8	5.27	0.0439	0.7
S_7_	ππ* C8 _+G8′_	5.31	0.3352	0.2	ππ* C6 _+G6′_	5.28	0.1548	0.0
S_8_	CT G8- > hmC	5.37	0.0533	0.5	ππ* C8 _+CT_	5.33	0.1913	0.2

**Fig. 5:**
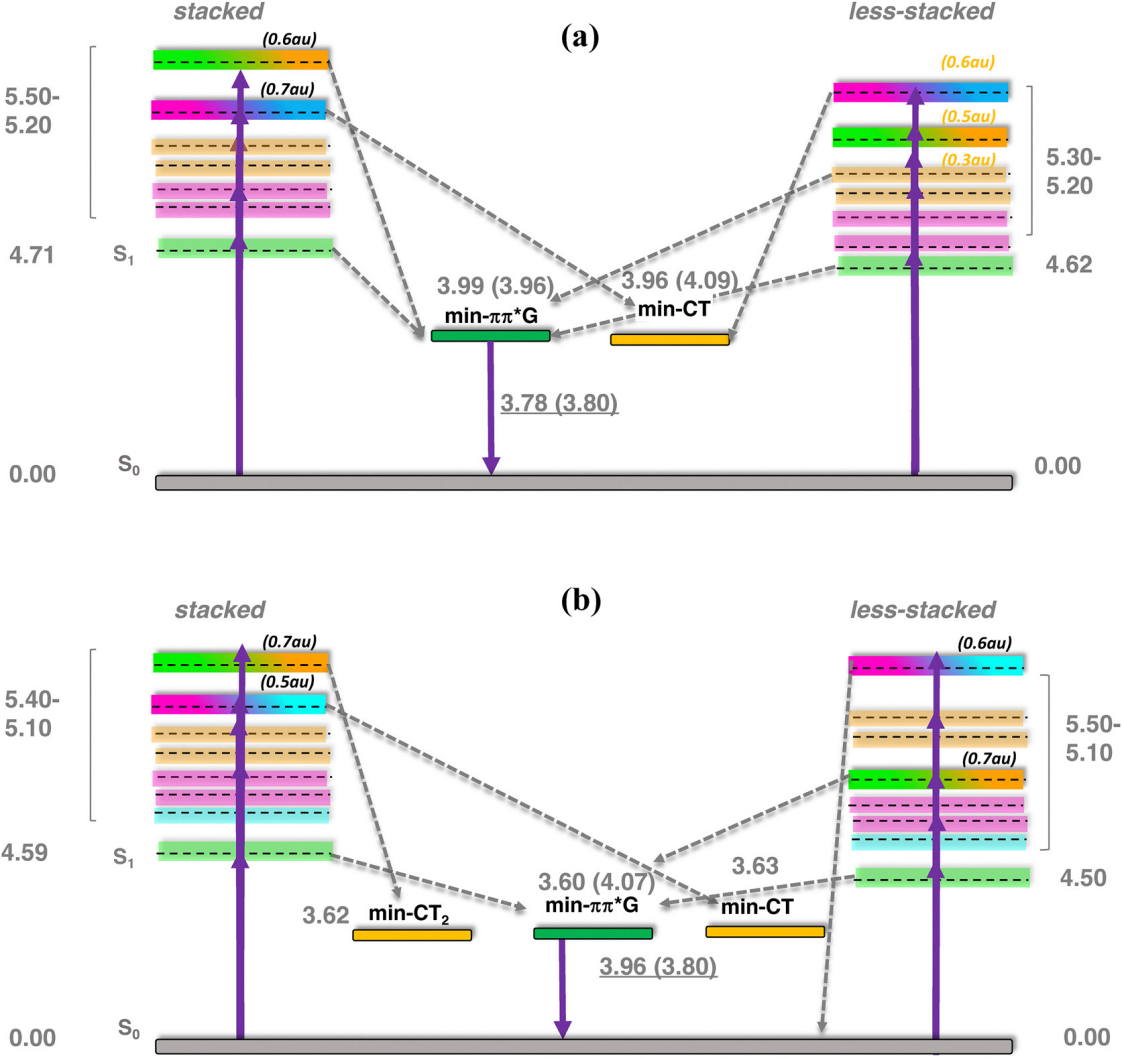
Excited state potential energy surface for C2ApC/GTG (a) and C2ApC/GhmCG (b). The Franck–Condon region is shown for both stacked (left) and less-stacked (right) cases, whereas the evolution towards the minima is shown in the middle. Adiabatic energies (respect to the ground state at Franck–Condon) and emission energies underlined (in eV) for both stacked and less-stacked (parenthesis) systems.

To further understand the fluorescence emission energies and yields, the S_1_ bright excited state and the first two states with significant CT character (>0.3 a.u.) were optimized for the four systems. The S_1_ state evolves towards a minimum in the first excited Potential Energy Surface (PES) for all of them (Fig. 5 and Table S6). The minimum, that is located on the 2Ap base presents emission energies in the 3.70–3.80 eV energy range, that correspond with Stokes shifts of ∼0.8–0.9 eV, except for the hmC less-stacked system whose emission energy is almost 4 eV (blue shifted, Stokes shift ∼0.54 eV), in line with experimental trends (Stokes shift 0.56 eV, for C2ApC/GhmCG Fig. 4b). This latter minimum resembles the minimum for the isolated 2Ap base in solution that we have also optimized for comparison, and has an emission energy of 4.04 eV.

The reason for this difference is that for the other three systems, hmC-stacked and T-stacked or T-less-stacked, the minima, even if localized on 2Ap, have a small CT character with the adjacent C6 base (0.1–0.2 a.u., Table S6). This partial CT character is also reflected in the lower oscillator strength (f) of those minima, being at a maximum of 0.2, compared to hmC-less-stacked and isolated 2Ap, where it amounts to 0.3. If only this minimum would be present, and if we assume that the non-radiative decays are the same, hmC-less stacked would be more emissive compared to the rest of the systems. Then, for all the systems, at least one minimum with strong CT character was located. This is in agreement with the quenching of 2Ap fluorescence within DNA observed experimentally (Fig. 4 and Table 2). In hmC-stacked the CT is so strong as observed in absorption that when the S_11_ is optimized the system evolves to a degeneracy region with the ground state, in which a bond is created between hmC and G. In the less-stacked system, the CT is not so strong and, although slightly, the CT minima are less stable than the bright ones. In T-stacked and less-stacked the CT minimum involving the G8-T is as stable as the bright one.

**Table 2:** Decay-associated spectral properties, rate constants and QY of 2Ap-labeled ssDNA, matched, mismatched and modified dsDNA sequences

Sample	*τ*_1_ (ns)	*A*_1_	*f*_1_	*τ*_2_ (ns)	*A*_2_	*f*_2_	*τ*_3_ (ns)	*A*_3_	*f*_3_	*τ*_av_ (ns)	*k*_r_ (10^7^ × s^−1^)	*k*_nr_ (10^7^×s^−1^)	QY
C2ApC	0.61	0.53	0.09	2.35	0.21	0.14	10.00	0.27	0.76	3.47	0.49	28.34	0.0173
C2ApC/GTG	0.61	0.21	0.03	2.35	0.32	0.15	10.00	0.42	0.83	5.12	0.09	19.45	0.0049
C2ApC/GCG	0.61	0.25	0.03	2.35	0.32	0.14	10.00	0.44	0.83	5.27	0.11	18.87	0.0063
C2ApC/GmCG	0.61	0.24	0.03	2.35	0.33	0.15	10.00	0.43	0.82	5.18	0.11	19.19	0.0059
C2ApC/GhmCG	0.61	0.24	0.03	2.35	0.31	0.14	10.00	0.45	0.83	5.35	0.18	18.50	0.0100

### 3.3. Excited state dynamics impacted by epigenetic bases in DNA

#### 3.3.1. Time-resolved fluorescence and decay-associated spectral analysis

The fluorescence decay of both matched and mismatched DNA duplexes is shown at a maximum emission wavelength of 370 nm (Fig. S9). Rather than focusing on a particular emission maximum wavelength, we investigated the full emission range utilizing the DAS spectra, which allows for the segregation of lifetime components and provides insights into molecular conformational alteration.^56,67^ Fluorescence decay measurements were recorded for the 2Ap-labeled matched and mismatched DNA duplexes across the 320–430 nm emission range with 10 nm intervals to generate the decay-associated spectra, DAS (Table 2). The 2Ap fluorescence decay was well-fitted with three lifetime components (Table S7) for all matched and mismatched duplexes, each correlated with a specific conformational state. ssDNA has a lower average lifetime compared to dsDNA, observed because the fluorescence lifetime of 2Ap varies based on its position within the ODN, with time constants ranging from ∼50 ps to 10 ns.^36,37,62,68,69^ The high amplitude populations (*α*_1_) are associated with the lifetime (*τ*_1_), which corresponds to the most strongly stacked conformations of 2Ap. Based on these high percentages, it is clear that 2Ap contributes to stacking interactions with neighbouring bases in the duplex, also suggesting that 2Ap has minimal impact on the structure of the duplex. As a result, the 2Ap residue is stacked efficiently within the duplex. The long lifetimes (*τ*_2_ and *τ*_3_) are likely a result of unstacked or extrahelical conformations.

The amplitude value of *A*_*i*_ was determined by integrating areas under the DAS profile of each lifetime component, normalized to the total steady-state fluorescence intensity. The fractional intensity values (*f*_*i*_) were calculated by 
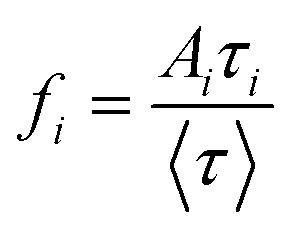
. The non-radiative and radiative rate constants were determined by 
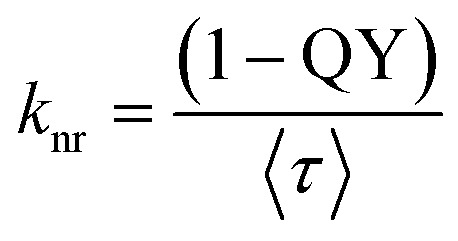
 and 
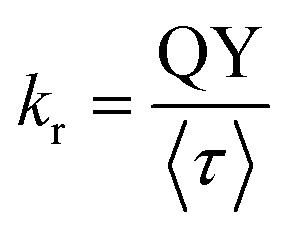
 respectively. In DAS experiments, the average error was ≤ 5%.

**Fig. 6:**
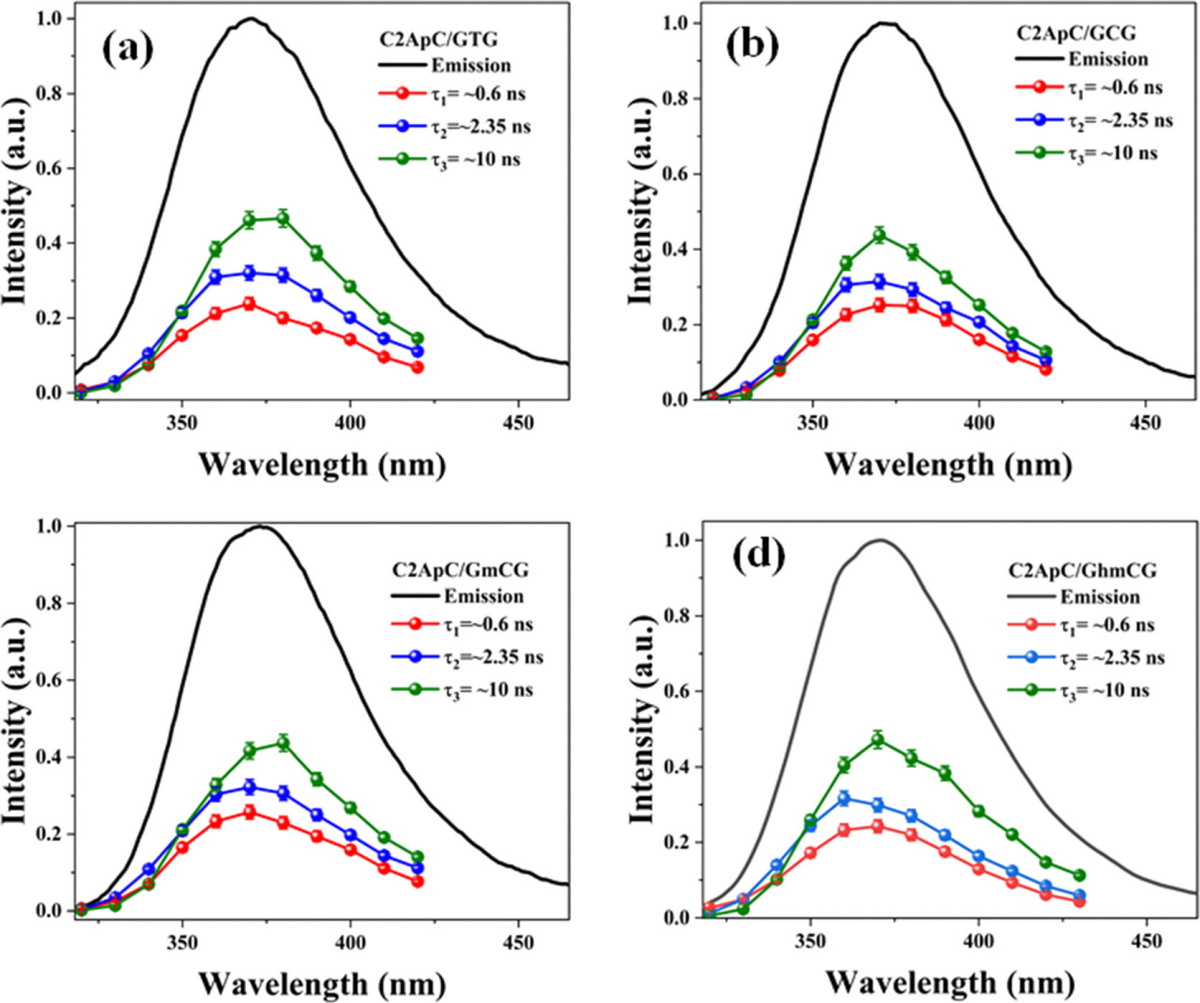
DAS spectra of matched, mismatched, and modified dsDNA duplexes. The black lines represent the normalized steady-state fluorescence spectra.

As represented in Fig. S10 and Table 2, the DAS spectra of ssDNA exhibited a high contribution from the *τ*_1_ lifetime component relative to the steady-state emission profile. Furthermore, significant contributions from three lifetime components are witnessed across the entire emission profile. Nonetheless, a clear differentiation is observed in the *τ*_2_ and *τ*_3_ components, with the longer lifetime component (*τ*_3_) predominantly seeming on the red side of the emission spectrum. As represented in Table 2 and Fig. 6, the amplitudes of individual lifetime components show minimal differences between the matched and mismatched duplexes. The *τ*_3_ lifetime component exhibits a significantly superior amplitude contribution across the entire steady-state emission spectrum associated with the *τ*_2_ and *τ*_1_ components in the 2Ap-labeled dsDNA duplex. However, the average lifetime values from the DAS spectra show a slight difference, with C2ApC/GhmCG exhibiting an *τ*_av_ of 5.35 ns compared to 5.12 ns for C2ApC/GTG. It is interesting to note that the QY increase in the hmC clearly correlated with the slight increase in the (*α*_3_) population.

This suggests a significant contribution of segmental motions, indicating the high flexibility of the duplexes. The non-radiative (*k*_nr_) and radiative (*k*_r_) rate constants were derived through an evaluation of the QY and the average excited-state lifetimes (*τ*_av_). Since *k*_r_ values are relative to the extinction coefficients of the chromophores,^39^ the reduced *k*_r_ values align with the hypochromism witnessed in the absorption spectra. Furthermore, our data show that the slightly higher QY values of hmC duplexes, compared to thymine duplexes, are primarily due to decreased *k*_nr_. This confirms the decrease in additional non-radiative pathways in hmC modified duplexes compared to matched duplexes. The *k*_nr_ values (Table 2) exhibit a strong correlation with the stacking behaviour observed in MD simulations (Fig. 3). Precisely, the two systems with reduced stacking interactions (hmC and C) correspond to those with the lowest *k*_nr_ values.

## Discussion

Epigenetic modifications, such as methylation and demethylation, play a crucial role in regulating DNA structure and function. Accurately probing the structural dynamics of modified nucleobases within these pathways is essential for understanding their biochemical impact. 2Ap, a fluorescent adenine analogue with high environmental sensitivity, serves as a valuable probe for detecting local conformational changes in DNA. However, a deeper understanding of its photophysical behaviour within nucleic acids is needed to expand its applications in studying modified DNA. This study integrates experimental and theoretical approaches to examine the structural and spectral effects of methylation, hydroxymethylation, and demethylation in DNA duplexes, with 2Ap strategically positioned to report on local changes.

The fluorescence properties of 2Ap in DNA provide a deep insight into the structural dynamics, conformational fluctuations, and interaction mechanisms of DNA and RNA.^69–72^ Its fluorescence behaviour encompassing intensity, lifetime, and decay kinetics is intricately modulated by its immediate nucleic acid environment. A major observation is the significant quenching of 2Ap fluorescence in dsDNA compared to ssDNA, which occurs primarily due to base stacking interactions and the restriction of the excited-state dynamics of 2Ap within the duplex.^31,34,37,69,71^ This quenching mechanism is driven by both dynamic (collisional) and static (ground-state complex) interactions between 2Ap and neighbouring nucleotides, with varying contributions depending on the sequence context and structural arrangement of the DNA.^31,34,37,69,71^ This quenching is further influenced by charge-transfer interactions, as demonstrated in studies of 2Ap paired with a mismatched base pair (cytosine), and by theoretical studies^73,74^ (see below), where an equilibrium exists between neutral wobble and protonated Watson–Crick configurations.^75^ 2Ap fluorescence QYs are sensitive to base stacking, with mismatched duplex DNA exhibiting higher yields than well-matched duplexes, and ssDNA showing the highest yields due to the absence of base stacking.^36^ Furthermore, its fluorescence lifetime also varies depending on its position within the ODN, with time constants ranging from ∼50 ps to 10 ns.^36,37,62,68^ In short, the photophysical properties of 2Ap fluorescence are influenced by DNA sequence context, making 2Ap an effective probe for distinguishing variations in DNA stability and structural integrity. Methylation of cytosines at specific positions in 2Ap-labeled dsDNA can destabilize neighbouring residues to the same extent, leading to increased fluorescence QYs and longer fluorescence lifetimes.^36^ This sequence-dependent modulation underscores the sensitivity of 2Ap fluorescence to local DNA conformations and interactions.

Base stacking interactions stabilize our here studied 2Ap-labeled matched DNA duplex (C2ApC/GTG) by promoting a highly ordered conformation, in view of the results of the thermal, CD spectra, and MD simulations (Fig. 2 and 3). This is manifested as reduced absorbance compared to ssDNA (Fig. 4a). However, both MD and absorption measurements (larger intensity compared to matched duplex) demonstrate that mismatched duplexes disrupt to some extent this stacking, but keeping the global dsDNA structure, with special attention to C2ApC/GCG and C2ApC/GhmCG (Fig. 2). Notably, the C2ApC/GhmCG duplex exhibits a slight increase in ellipticity at 290 nm compared to C2ApC/GCG and C2ApC/GmCG, suggesting that hmC induces subtle structural changes (Fig. 2b). This could reflect an alteration in helical conformation or base stacking, likely influenced by the hydroxymethyl group at the 5-position of cytosine. Such modification could impact base stacking and hydrogen bonding interactions, leading to a structurally distinct DNA duplex. These findings suggest that hmC may alter the DNA's local secondary structure, potentially influencing its flexibility and base-pairing properties, providing further insight into the structural dynamics of modified cytosine-containing duplexes. This intensity dependence of the low energy region of the spectra on the nature of the X bases is also captured by the oscillator strength (Table 1) for the S_1_ ππ* excited state. Indeed, the C2ApC/GTG stacked system is 10% smaller than the C2ApC/GhmCG less stacked one. Despite these differences in intensities and the energies of the lowest lying state, the global maximum is not very much affected by the inclusion of the epigenetic bases (Fig. S7). On the other hand, the fluorescence intensities of ssDNA and dsDNA variants differ significantly (Fig. 4), with ssDNA showing higher intensity due to less base stacking. Mismatched duplexes exhibit increased fluorescence compared to the matched duplexes. A blue shift in emission spectra (Fig. S6) further suggests that cytosine modifications, especially hmC, influence duplex conformation and stability. Such spectral shifts indicate changes in base stacking interactions and DNA conformation, resulting in a more hydrophobic environment with a lower dielectric constant around 2Ap.^76^

**Scheme 2:**
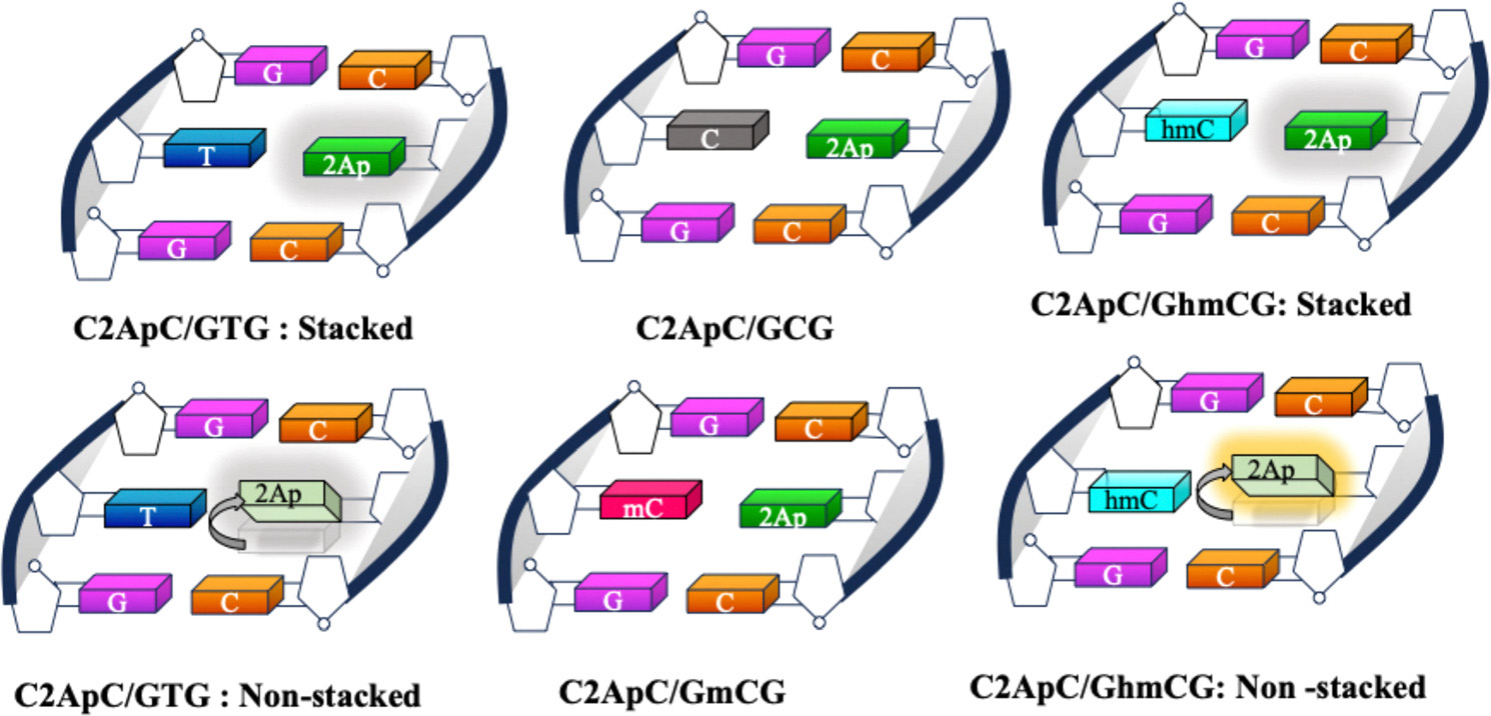
Schematic representation of stacked and non-stacked confirmations for matched, mismatched, methylated, and hydroxymethylated duplexes.

In the same line, 2Ap-labeled dsDNA shows markedly reduced QYs compared to free 2Ap. For instance, ssDNA (C2ApC) exhibits a QY of 0.0173, while the matched duplex (C2ApC/GTG) is at 0.0049 due to enhanced base stacking (Table 2). Comparable QYs in C2ApC/GCG and C2ApC/GmCG contrast with the higher QY (0.01) of C2ApC/GhmCG. It should be noted that C2ApC/GhmCG is the system that also displays the larger radiative rate (*k*_r_), which leads to a significant increase in QY. Focussing on the non-radiative rate (*k*_nr_), which is more directly related to the stacking, C2ApC/GhmCG also displays the lower rate, again consistent with the lesser stacking. In this case, it is also worth mentioning that the trend of *k*_nr_ values correlates quite well with the stacking behaviour observed in the MD simulations (Fig. 3), with hmC and C, which display larger *k*_nr_, being the system with larger deviations from perfect stacking. QM results support these findings, revealing that all the studied systems present two kinds of excited minima: a bright ππ* minimum localized on 2Ap responsible for the fluorescence, plus a CT minimum responsible for the fluorescence quenching compared to the free 2Ap. This fluorescence quenching due to the presence of CT states between 2Ap and adjacent bases has already been reported in the literature for reduced DNA models, such as dinucleotides or trimers.^73,74^ Other potential quenching mechanisms occurring through CI or exciplexes have also been characterized in the literature.^74,77,78^ Our increased ds hexamer model allows not only to see how efficient these CT minima are in the duplex but to estimate how different CT pathways compete. Indeed, we observe two coupled effects that may affect the fluorescence quenching. On one hand, the bright ππ* 2Ap minimum has some degree of 2amP- >C6 CT, except for the C2ApC/GhmCG less-stacked system (Table S6 and Scheme 2). Interestingly, this duplex is the one showing the larger fluorescence yield (Table 2). On the other hand, the main quenching effect should be due to the loss of excited state population towards G8- >T/hmC CT minima, which *a priori* are not directly related to 2Ap. Although both intra and interstrand CT states are present in the FC region, we have not located signs for proton transfer reactions as in canonical DNA.^79–81^ It must be noted that we have not attempted to locate ππ* minima localized on hmC or mC since those excited states have been characterized to decay in the ps timescale, that is shorter than our ns lifetimes,^82,83^ due to the accessible S_1_/S_0_ CI.^29,83,84^ Other epigenetic bases, such as 5-formyl-cytosine, do survive longer but are prone to populate triplet states,^85,86^ which is out of the scope of the present paper. Lastly, TCSPC analysis revealed that the matched and mismatched duplexes show similar amplitude distributions; however, the C2ApC/GhmCG duplex exhibits a slightly longer average lifetime and an enhanced long-lifetime (*α*_3_) population. This suggests that hmC may destabilize neighbouring residues, reducing non-radiative decay pathways. Consequently, the lower *k*_nr_ value in C2ApC/GhmCG duplexes correlates with their modestly increased QY, highlighting the sensitivity of 2Ap fluorescence to subtle conformational and dynamic variations in DNA.

## Conclusions

Herein, we unveiled the structural and dynamic impact of the epigenetic markers on DNA with the help of an intrinsic fluorescent nucleobase analogue, 2-aminopurine (2Ap), combined with molecular dynamics and quantum mechanical calculations. To the best of our knowledge, this study provides the first comprehensive characterization of the photophysical properties of 2Ap within dsDNA through a combined multi-technique experimental and multi-level computational approach. The results demonstrate that while all 2Ap-labeled duplexes exhibit significant base stacking interactions, keeping the overall structure of dsDNA, subtle variations in stacking degrees occur as a function of the epigenetic base. Indeed, our findings reveal that hmC modifications modulate local DNA conformation by diminishing base stacking and helical stability without compromising overall duplex integrity. This structural modulation results in a more hydrophobic environment around 2Ap, as indicated by blue-shifted emission spectra. Although CT-induced quenching is present in all systems, hmC shows reduced CT character compared to thymine, leading to enhanced fluorescence QYs, although still small, and longer lifetimes. These insights underscore the sensitivity of 2Ap fluorescence to subtle DNA conformational changes and suggest that hmC could play a regulatory role in biological processes by influencing DNA dynamics and local stability. This work not only expands the utility of 2Ap as an environmental probe but also highlights the potential epigenetic implications of hmC in modulating DNA structural and photophysical properties, opening the route for further studies with even more sensitive probes. Such modulation of DNA dynamics offers important insights into the role of epigenetic modifications in cellular function and provides a potential avenue for understanding the molecular underpinnings of epigenetic regulation.

## Conflicts of interest

There are no conflicts to declare.

## Supplementary Material

CB-OLF-D5CB00207A-s001

## Data Availability

The data supporting this article have been included as part of the supplementary information (SI) and the results of the molecular dynamics simulations have been uploaded to DOI: 10.5281/zenodo.17312412. Supplementary information contains MD details and results as well as more excited state analysis from the QM calculations and further experimental results. See DOI: https://doi.org/10.1039/d5cb00207a.

## References

[cit1] Carter B., Zhao K. (2021). Nat. Rev. Genet..

[cit2] Allis C. D., Jenuwein T. (2016). Nat. Rev. Genet..

[cit3] Kriukienė E., Tomkuvienė M., Klimašauskas S. (2024). Chem. Soc. Rev..

[cit4] Tahiliani M., Koh K. P., Shen Y., Pastor W. A., Bandukwala H., Brudno Y., Agarwal S., Iyer L. M., Liu D. R., Aravind L. (2009). Science.

[cit5] Ito S., Shen L., Dai Q., Wu S. C., Collins L. B., Swenberg J. A., He C., Zhang Y. (2011). Science.

[cit6] Smith Z. D., Meissner A. (2013). Nat. Rev. Genet..

[cit7] Bestor T. H., Ingram V. M. (1983). Proc. Natl. Acad. Sci. U. S. A..

[cit8] Cooper D. N., Youssoufian H. (1988). Hum. Genet..

[cit9] Jones P. A. (2012). Nat. Rev. Genet..

[cit10] Johnson T. B., Coghill R. D. (1925). J. Am. Chem. Soc..

[cit11] Holliday R., Pugh J. E. (1975). Science.

[cit12] Dahl C., Guldberg P. (2003). Biogerontology.

[cit13] Cannistraro V. J., Taylor J.-S. (2009). J. Mol. Biol..

[cit14] Jin S.-G., Xiong W., Wu X., Yang L., Pfeifer G. P. (2015). Genomics.

[cit15] Denissenko M. F., Chen J. X., Tang M.-S., Pfeifer G. P. (1997). Proc. Natl. Acad. Sci. U. S. A..

[cit16] Rideout W. M., Coetzee G. A., Olumi A. F., Jones P. A. (1990). Science.

[cit17] Drouin R., Therrien J.-P. (1997). Photochem. Photobiol..

[cit18] Stroud H., Feng S., Morey Kinney S., Pradhan S., Jacobsen S. E. (2011). Genome Biol..

[cit19] Battistini F., Dans P. D., Terrazas M., Castellazzi C. L., Portella G., Labrador M., Villegas N., Brun-Heath I., González C., Orozco M. (2021). PLoS Comput. Biol..

[cit20] Wang Y., Zhang X., Wu F., Chen Z., Zhou X. (2019). Chem. Sci..

[cit21] Zeng T., Liu L., Li T., Li Y., Gao J., Zhao Y., Wu H.-C. (2015). Chem. Sci..

[cit22] Lian C. G., Xu Y., Ceol C., Wu F., Larson A., Dresser K., Xu W., Tan L., Hu Y., Zhan Q. (2012). Cell.

[cit23] Iurlaro M., Ficz G., Oxley D., Raiber E.-A., Bachman M., Booth M. J., Andrews S., Balasubramanian S., Reik W. (2013). Genome Biol..

[cit24] Kellinger M. W., Song C.-X., Chong J., Lu X.-Y., He C., Wang D. (2012). Nat. Struct. Mol. Biol..

[cit25] Nakano S., Suzuki T., Kawarada L., Iwata H., Asano K., Suzuki T. (2016). Nat. Chem. Biol..

[cit26] Ngo T. T., Yoo J., Dai Q., Zhang Q., He C., Aksimentiev A., Ha T. (2016). Nat. Commun..

[cit27] You Y.-H., Pfeifer G. P. (2001). J. Mol. Biol..

[cit28] Ikehata H., Ono T. (2011). J. Radiat. Res..

[cit29] Martinez-Fernandez L., Banyasz A., Esposito L., Markovitsi D., Improta R. (2017). Sig. Transduct. Target. Ther..

[cit30] Dziuba D., Didier P., Ciaco S., Barth A., Seidel C. A., Mély Y. (2021). Chem. Soc. Rev..

[cit31] Jean J. M., Hall K. B. (2001). Proc. Natl. Acad. Sci. U. S. A..

[cit32] O'Neil M. A., Barton J. K. (2002). J. Am. Chem. Soc..

[cit33] Wan C., Fiebig T., Schiemann O., Barton J. K., Zewail A. H. (2000). Proc. Natl. Acad. Sci. U. S. A..

[cit34] Dallmann A., Dehmel L., Peters T., Mügge C., Griesinger C., Tuma J., Ernsting N. P. (2010). Angew. Chem., Int. Ed..

[cit35] Eritja R., Kaplan B. E., Mhaskar D., Sowers L. C., Petruska J., Goodman M. F. (1986). Nucleic Acids Res..

[cit36] Greiner V. J., Kovalenko L., Humbert N., Richert L., Birck C., Ruff M., Zaporozhets O. A., Dhe-Paganon S., Bronner C., Mely Y. (2015). Biochemistry.

[cit37] Jones A. C., Neely R. K. (2015). Q. Rev. Biophys..

[cit38] Kilin V., Gavvala K., Barthes N. P., Michel B. Y., Shin D., Boudier C., Mauffret O., Yashchuk V., Mousli M., Ruff M. (2017). J. Am. Chem. Soc..

[cit39] Lakowicz J. R. , Principles of fluorescence spectroscopy, Springer, 2006

[cit40] Knutson J. R., Beechem J. M., Brand L. (1983). Chem. Phys. Lett..

[cit41] Beechem J. M., Ameloot M., Brand L. (1985). Chem. Phys. Lett..

[cit42] Hanwell M. D., Curtis D. E., Lonie D. C., Vandermeersch T., Zurek E., Hutchison G. R. (2012). J. Cheminf..

[cit43] Ivani I., Dans P. D., Noy A., Pérez A., Faustino I., Hospital A., Walther J., Andrio P., Goñi R., Balaceanu A., Portella G., Battistini F., Gelpí J. L., González C., Vendruscolo M., Laughton C. A., Harris S. A., Case D. A., Orozco M. (2016). Nat. Methods.

[cit44] Wang J., Wang W., Kollman P. A., Case D. A. (2006). J. Mol. Graphics Modell..

[cit45] Chai J.-D., Head-Gordon M. (2008). Phys. Chem. Chem. Phys..

[cit46] Frisch M. J. , Trucks G. W., Schlegel H. B., Scuseria G. E., Robb M. A., Cheeseman J. R., Scalmani G., Barone V., Petersson G. A., Nakatsuji H., Li X., Caricato M., Marenich A. V., Bloino J., Janesko B. G., Gomperts R., Mennucci B., Hratchian H. P., Ortiz J. V., Izmaylov A. F., Sonnenberg J. L., Williams, Ding F., Lipparini F., Egidi F., Goings J., Peng B., Petrone A., Henderson T., Ranasinghe D., Zakrzewski V. G., Gao J., Rega N., Zheng G., Liang W., Hada M., Ehara M., Toyota K., Fukuda R., Hasegawa J., Ishida M., Nakajima T., Honda Y., Kitao O., Nakai H., Vreven T., Throssell K., Montgomery Jr. J. A., Peralta J. E., Ogliaro F., Bearpark M. J., Heyd J. J., Brothers E. N., Kudin K. N., Staroverov V. N., Keith T. A., Kobayashi R., Normand J., Raghavachari K., Rendell A. P., Burant J. C., Iyengar S. S., Tomasi J., Cossi M., Millam J. M., Klene M., Adamo C., Cammi R., Ochterski J. W., Martin R. L., Morokuma K., Farkas O., Foresman J. B. and Fox D. J., Gaussian 16, Wallingford, CT, 2016

[cit47] Pronk S., Páll S., Schulz R., Larsson P., Bjelkmar P., Apostolov R., Shirts M. R., Smith J. C., Kasson P. M., van der Spoel D., Hess B., Lindahl E. (2013). Bioinformatics.

[cit48] Nosé S. (1984). Mol. Phys..

[cit49] Hoover W. G. (1985). Phys. Rev. A..

[cit50] Parrinello M., Rahman A. (1981). J. Appl. Phys..

[cit51] Darden T., York D., Pedersen L. (1993). J. Chem. Phys..

[cit52] Hess B., Bekker H., Berendsen H. J. C., Fraaije J. G. E. M. (1997). J. Comput. Chem..

[cit53] Mignani S., Rosa R. (1995). Comput. Phys. Commun..

[cit54] Zhao Y., Truhlar D. G. (2008). Acc. Chem. Res..

[cit55] Zhao Y., Schultz N. E., Truhlar D. G. (2006). J. Chem. Theory Comput..

[cit56] Kuchlyan J., Martinez-Fernandez L., Mori M., Gavvala K., Ciaco S., Boudier C., Richert L., Didier P., Tor Y., Improta R. (2020). J. Am. Chem. Soc..

[cit57] Tomasi J., Mennucci B., Cammi R. (2005). Chem. Rev..

[cit58] Miertuš S., Scrocco E., Tomasi J. (1981). Chem. Phys..

[cit59] Markham N. R., Zuker M. (2005). Nucleic Acids Res..

[cit60] Johnson N. P., Baase W. A., Von Hippel P. H. (2004). Proc. Natl. Acad. Sci. U. S. A..

[cit61] Law S. M., Eritja R., Goodman M. F., Breslauer K. J. (1996). Biochemistry.

[cit62] Xu D.-G., Nordlund T. M. (2000). Biophys. J..

[cit63] Xu D., Evans K. O., Nordlund T. M. (1994). Biochemistry.

[cit64] Berg J. M. , Tymoczko J. L. and Stryer L., Biochemistry (loose-leaf), Macmillan, 2007

[cit65] Nordlund T. M., Xu D., Evans K. O. (1993). Biochemistry.

[cit66] Ward D., Reich E., Stryer L. (1969). J. Biol. Chem..

[cit67] Takkella D., Martinez-Fernandez L., Gavvala K. (2024). J. Photochem. Photobiol. A: Chem..

[cit68] Goel T., Mukherjee T., Rao B. J., Krishnamoorthy G. (2010). J. Phys. Chem. B.

[cit69] Ramreddy T., Rao B. J., Krishnamoorthy G. (2007). J. Phys. Chem. B.

[cit70] Ballin J. D., Bharill S., Fialcowitz-White E. J., Gryczynski I., Gryczynski Z., Wilson G. M. (2007). Biochemistry.

[cit71] Manoj P., Min C.-K., Aravindakumar C., Joo T. (2008). Chem. Phys..

[cit72] Sholokh M., Sharma R., Shin D., Das R., Zaporozhets O. A., Tor Y., Mély Y. (2015). J. Am. Chem. Soc..

[cit73] Jean J. M., Hall K. B. (2002). Biochemistry.

[cit74] Liang J., Nguyen Q. L., Matsika S. (2013). Photochem. Photobiol. Sci..

[cit75] Sowers L. C., Boulard Y., Fazakerley G. V. (2000). Biochemistry.

[cit76] Su T. J., Connolly B. A., Darlington C., Mallin R., Dryden D. T. (2004). Nucleic Acids Res..

[cit77] Liang J., Matsika S. (2011). J. Am. Chem. Soc..

[cit78] Liang J., Matsika S. (2012). J. Am. Chem. Soc..

[cit79] Martinez Fernandez L., Santoro F., Improta R. (2022). Acc. Chem. Res..

[cit80] Martinez-Fernandez L., Improta R. (2018). Faraday Discuss..

[cit81] Fernández L. M. and Improta R., Nucleic Acid Photophysics and Photochemistry, Springer, 2024, pp. 29–50

[cit82] Wang X., Yu Y., Zhou Z., Liu Y., Yang Y., Xu J., Chen J. (2019). J. Phys. Chem. B.

[cit83] Martínez-Fernández L., Pepino A., Segarra-Martí J., Jovaisaite J., Vaya I., Nenov A., Markovitsi D., Gustavsson T., Banyasz A., Garavelli M. (2017). J. Am. Chem. Soc..

[cit84] Kabacinski P., Romanelli M., Ponkkonen E., Jaiswal V. K., Carell T., Garavelli M., Cerullo G., Conti I. (2021). J. Phys. Chem. Lett..

[cit85] Wang X., Martínez-Fernández L., Zhang Y., Wu P., Kohler B., Improta R., Chen J. (2024). J. Am. Chem. Soc..

[cit86] Wang X., Martínez-Fernández L., Zhang Y., Zhang K., Improta R., Kohler B., Xu J., Chen J. (2021). Chem. – Eur. J..

