# Supplementary material for: New insights into the structure and dynamics of the epigenetic modifications on DNA
Source: RSC Chem Biol. 2025 Oct 13;6(12):1927–40. doi: 10.1039/d5cb00207a (PMC12539948; doi:10.1039/d5cb00207a)
Supplement: CB-006-D5CB00207A-s001 [file CB-006-D5CB00207A-s001.pdf]

Supplementary Information (SI) for RSC Chemical Biology.
This journal is © The Royal Society of Chemistry 2025

Supporting Information

New Insights into the Structure and Dynamics of the Epigenetic Modifications on DNA

Dineshbabu Takkella[1], Javier Cerezo[2], Lara Martinez-Fernandez [3,*], Krishna Gavvala[1,*]

[1]Department of Chemistry, Indian Institute of Technology Hyderabad, Kandi, Sangareddy, Telangana-502284, India.

[2]Departamento de Química, Facultad de Ciencias and Institute for Advanced Research in Chemistry (IADCHEM), Universidad Autónoma de Madrid, Campus de Excelencia UAM-CSIC, Cantoblanco, 28049 Madrid, Spain.

[3]Departamento de Química Física de Materiales, Instituto de Química Física Blas Cabrera, Consejo Superior de Investigaciones Científicas, IQF-CSIC, Calle Serrano 119, 28006, Madrid, Spain.

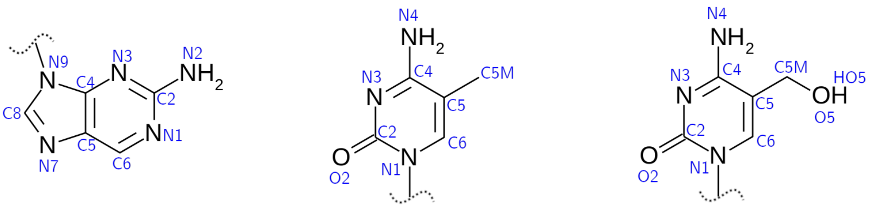

2Ap **mC** **hmC**

Scheme S1. Atom names for non-standard residues.

Table S1. Atom types assignment and charge for 2Ap. Atom types are taken from parmbsc1 force field.

2Ap				mC				hmC		
Atom	AtomType	Charge		Atom	AtomType	Charge		Atom	AtomType	Charge
N9	N*	-0.1168		N1	N*	-0.1403		N1	N*	-0.1718
C8	C1	0.2451		C6	CM	-0.1440		C6	CM	-0.0146
H8	H5	0.1294		H6	H4	0.2596		H6	H4	0.2195
N7	NB	-0.6136		C5	CM	-0.1229		C5	CM	-0.2530
C5	CB	0.0519		C5M	CT	-0.3377		C5M	CT	0.1568
C6	CA	0.2022		H5M1	HC	0.1162		H5M1	H1	0.0431
H6	H4	0.1211		H5M2	HC	0.1162		H5M2	H1	0.0431
N1	NC	-0.7146		H5M3	HC	0.1162		O5	OH	-0.5638
C2	CA	0.9219		C4	CA	0.6588		HO5	HO	0.4105
N2	N2	-0.9093		N4	N2	-0.7931		C4	CA	0.6381
H21	H	0.4075		H41	H	0.3960		N4	N2	-0.7655
H22	H	0.4075		H42	H	0.3960		H41	H	0.3816
N3	NC	-0.7348		N3	NC	-0.7884		H42	H	0.3816
C4	CB	0.3976		C2	C	0.7838		N3	NC	-0.7676
C3'	CE	0.0713		O2	O	-0.6619		C2	C	0.7797
H3'	H1	0.0985		C3'	CE	0.0713		O2	O	-0.6595
C2'	CT	-0.0854		H3'	H1	0.0985		C3'	CE	0.0713
H2'1	HC	0.0718		C2'	CT	-0.0854		H3'	H1	0.0985
H2'2	HC	0.0718		H2'1	HC	0.0718		C2'	CT	-0.0854
O3'	OS	-0.5232		H2'2	HC	0.0718		H2'1	HC	0.0718
				O3'	OS	-0.5232		H2'2	HC	0.0718
								O3'	OS	-0.5232

Table S2. Additional bonded parameters added to parmbsc1 force field to describe interactions within non-standard residues.

Angles; $V = k_\theta (\theta - \theta_0)$			Dihedrals; $V = k(1-\cos(2\phi - \phi_0)$		
Atom Types	θ_0 (deg)	k (kJ mol^{-1} rad^{-2})	Atom Types	ϕ_0 (deg)	k (kJ mol^{-1})
NC-CA-NC	129.1	585.76	NC-NC-CA-N2	180	4.6024
H4-CA-NC	115.45	418.4	CB-H4-CA-NC	180	4.6024
CM-CT-OH	109.5	418.4			
CA-NC-CA	118.6	585.76			

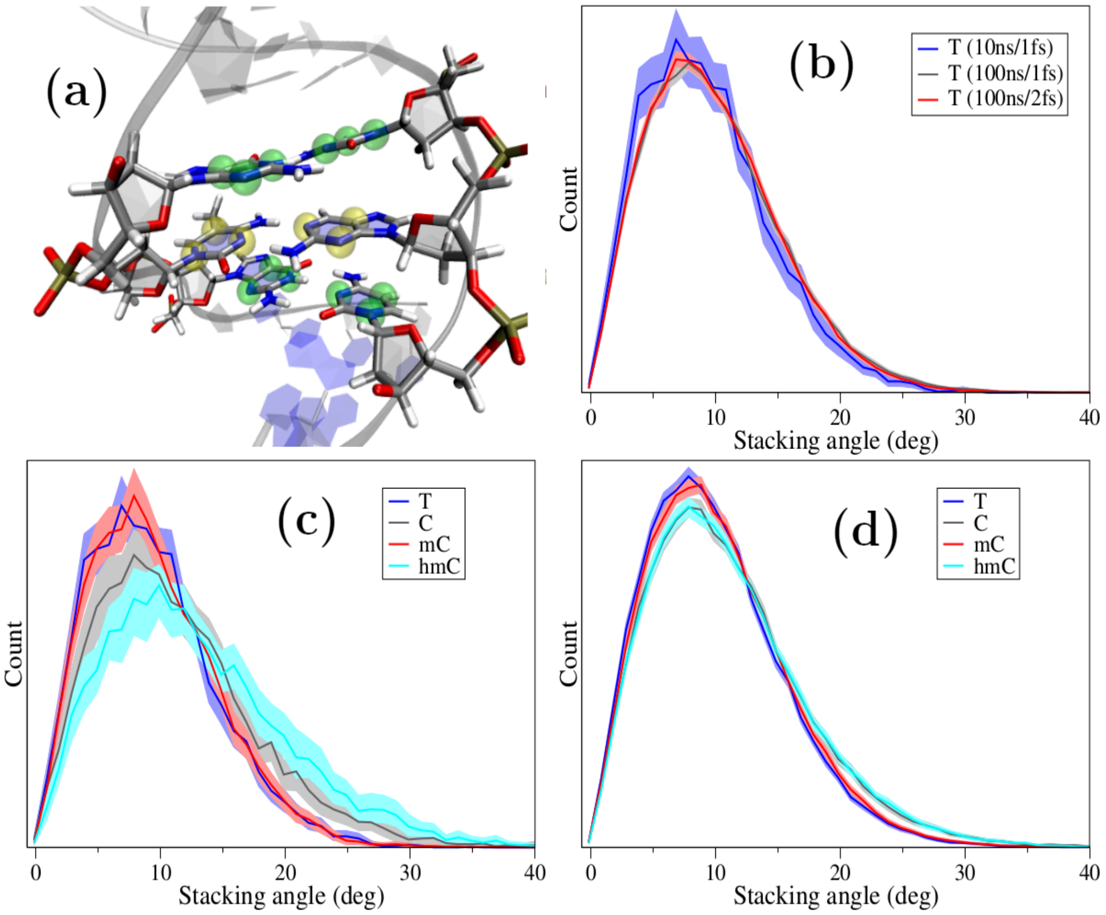

Figure S1. (a) Identification of the atoms that define the molecular planes used to compute the stacking angles among subsequent bases. In all cases, the 3 non-consecutive sites in the ring, excluding the C-NH_2, are selected. (b) Histogram of the stacking between 2Ap and C8 nucleobases for 2Ap and C8 nucleobases for T along the MD trajectories with different time steps and initial conditions. The average stacking from these distributions is 9.3º (10ns/1fs), 10.0º (100ns/1fs), 9.9º (100ns/2fs). (c) Histogram of the stacking between 2Ap and C8 nucleobases for all the X bases along the MD trajectories propagated for 10 ns. The average stacking from these distributions is 9.3º (T), 11.2º (C), 9.4º (mC) and 12.7º (hmC). (d) Histogram of the stacking between 2Ap and C8 nucleobases for all the X bases along the MD trajectories propagated for 100 ns. The average stacking from these distributions is 10.0º (T), 11.0º (C), 10.2º (mC) and 11.1º (hmC).

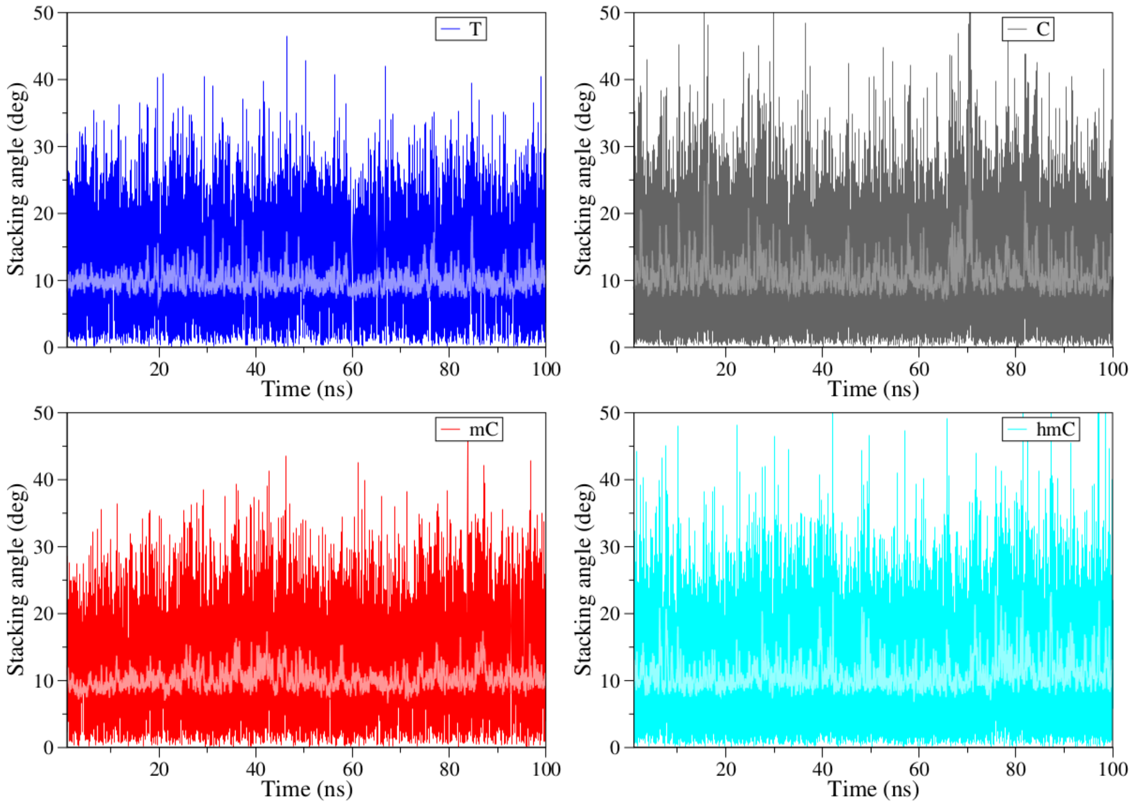

Figure S2. Evolution of the stacking degree between 2Ap and C8 for all the X residues along the MD simulations between the specified bases. Light-colored lines indicate the running average over 100 ps.

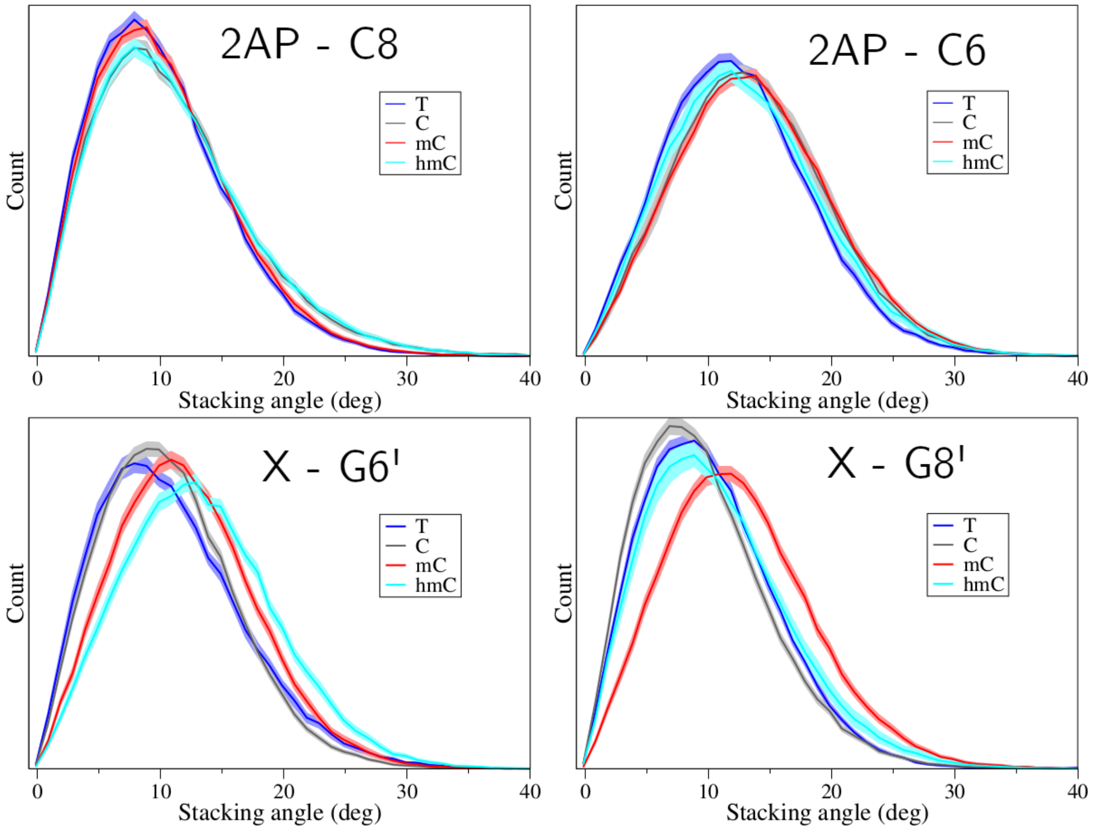

Figure S3. Histogram of the stacking between 2Ap and the neighboring bases (C8 and C6) and between the modified base (X=T, C, mC, hmC) and the neighboring bases (G8' and G6'), computed for all system over a MD trajectory of 100 ns.

Table S3. Melting temperature (T_m) values of the matched and mismatched 2Ap-labeled dsDNA sequences.

dsDNA	T_m (ºC)
C2ApC/GTG	59.47 ± 0.14
C2ApC/GCG	50.11 ± 0.17
C2ApC/GmCG	50.55 ± 0.18
C2ApC/GhmCG	50.9 ± 0.22

Table S4. Melting temperature (T_m) values of DNA duplexes, including those with a template guanine paired with C, mC, or hmC on the complementary strand.

dsDNA	T_m (ºC)
CGC/GCG	60.4 ± 0.4
CGC/GmCG	57.9 ± 0.5
CGC/GhmCG	48.5 ± 0.9

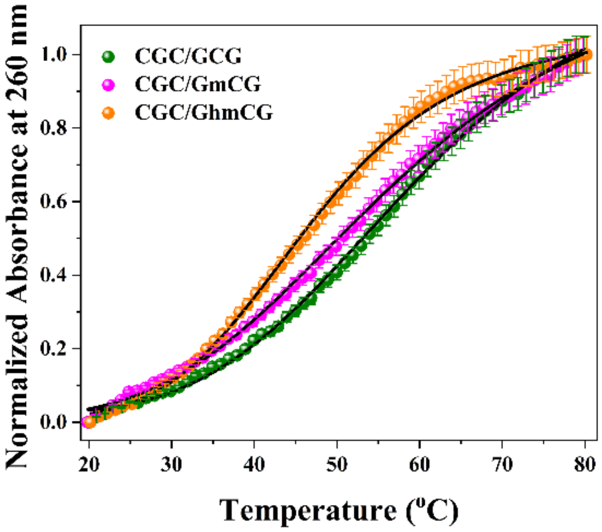

Figure S4. Melting temperature curves of DNA duplexes containing a template guanine paired with C, mC, and hmC on the complementary strand (5′-GGGCCC**G**CAGGG-3′/5′-CCCTG**X**GGGCCC-3′, where X = C, mC, and hmC).

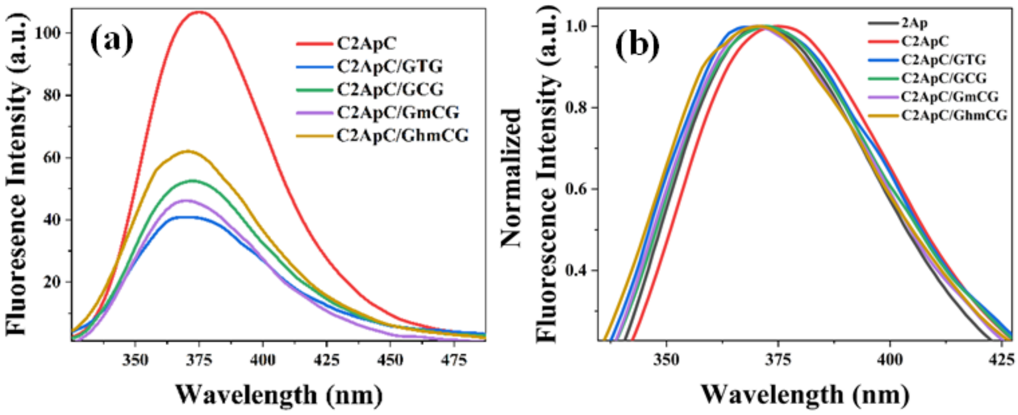

Figure S5. (a) Fluorescence emission and (b) normalized emission spectra of matched, mismatched and modified 2Ap-labeled dsDNA sequences. At 290 nm excitation wavelength.

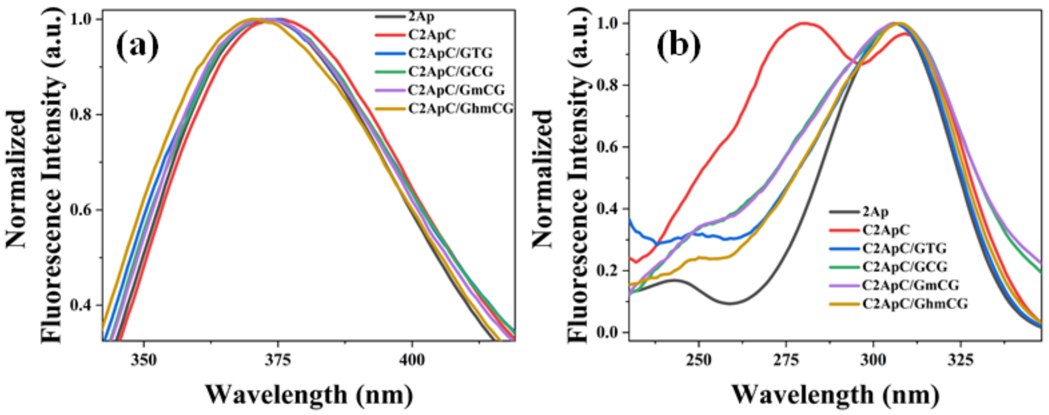

Figure S6. (a) Normalized fluorescence emission spectra of matched, mismatched and modified dsDNA sequences collected at 315 nm excitation wavelength. (b) Normalized fluorescence excitation spectra of respective dsDNA sequences collected at 370 nm emission wavelength.

Table S5. Excited state character, vertical absorption energies (in eV), oscillator strength and charge transfer character (CT, in a.u.) computed at the PCM/TD-M052X/6-31G(d) level of theory.

	Character	ΔE	f	CT	Character	ΔE	f	CT
	T-less stacked				T-stacked			
S_1	$\pi\pi^*$ 2aP	4.71	0.2187	0.0	$\pi\pi^*$ 2aP	4.62	0.1870	0.0
S_2	$n\pi^*$ 2aP	5.07	0.0052	0.0	$n\pi^*$ 2aP	5.06	0.0048	0.0
S_3	$n\pi^*$ T	5.17	0.0096	0.0	$n\pi^*$ T	5.17	0.0070	0.0
S_4	$\pi\pi^*$ G6’	5.18	0.0689	0.1	$\pi\pi^*$ G6’$_{+C6+C8}$	5.21	0.0968	0.1
S_5	$\pi\pi^*$ G8’	5.21	0.0613	0.1	$\pi\pi^*$ G8’$_{+C6+C8}$	5.21	0.1018	0.1
S_6	$\pi\pi^*$ C6	5.29	0.1663	0.1	$\pi\pi^*$ G8’+CT	5.24	0.0881	0.3
S_7	$\pi\pi^*$ C8$_{+C6}$	5.29	0.3268	0.1	$\pi\pi^*$ C6$_{+G6'}$	5.27	0.1788	0.1
S_8	CT G8->T	5.38	0.0538	0.7	CT 2aP->C8	5.32	0.1576	0.5
S_9	$\pi\pi^*$ T	5.43	0.1117	0.0	CT G8->T	5.35	0.0020	0.6
S_{10}	CT 2aP->C8	5.49	0.0701	0.6	$\pi\pi^*$ T	5.46	0.1273	0.1
	hmC-less stacked				hmC-stacked			
S_1	$\pi\pi^*$ 2aP	4.59	0.2119	0.0	$\pi\pi^*$ 2aP	4.50	0.1507	0.1
S_2	$n\pi^*$ 2aP	4.96	0.0035	0.0	$n\pi^*$ 2aP	4.95	0.0078	0.0
S_3	$\pi\pi^*$ hmC	5.06	0.1152	0.0	$\pi\pi^*$ hmC	5.06	0.0798	0.2
S_4	$\pi\pi^*$ G6’$_{+C6}$	5.23	0.0995	0.1	$\pi\pi^*$ G6’$_{+C6}$	5.21	0.1498	0.1
S_5	$\pi\pi^*$ G8’$_{+C8}$	5.26	0.0408	0.1	$\pi\pi^*$ G8’$_{+C8}$	5.25	0.0796	0.0
S_6	$\pi\pi^*$ C6 $_{+G6'}$	5.30	0.1478	0.0	CT 2aP->C8	5.27	0.0439	0.7
S_7	$\pi\pi^*$ C8 $_{+G8'}$	5.31	0.3352	0.2	$\pi\pi^*$ C6 $_{+G6'}$	5.28	0.1548	0.0
S_8	CT G8->hmC	5.37	0.0533	0.5	$\pi\pi^*$ C8 $_{+CT}$	5.33	0.1913	0.2
S_9	CT 2aP->C8	5.38	0.0172	0.7	CT 2aP->C6	5.35	0.0797	0.6
S_{10}	CT 2aP->G6’	5.46	0.0146	0.8	CT G6’->C6/2amP	5.42	0.0043	0.8
S_{11}					CT G8->hmC	5.48	0.0838	0.6

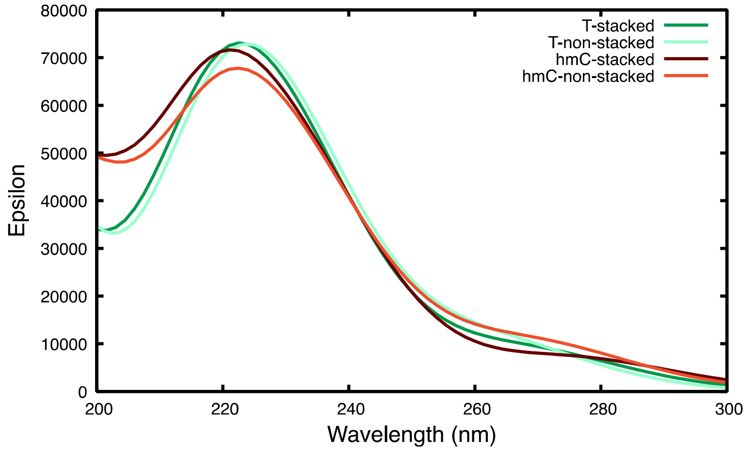

Figure S7. Absorption spectra computed for the four systems at the TD-DFT level of theory.

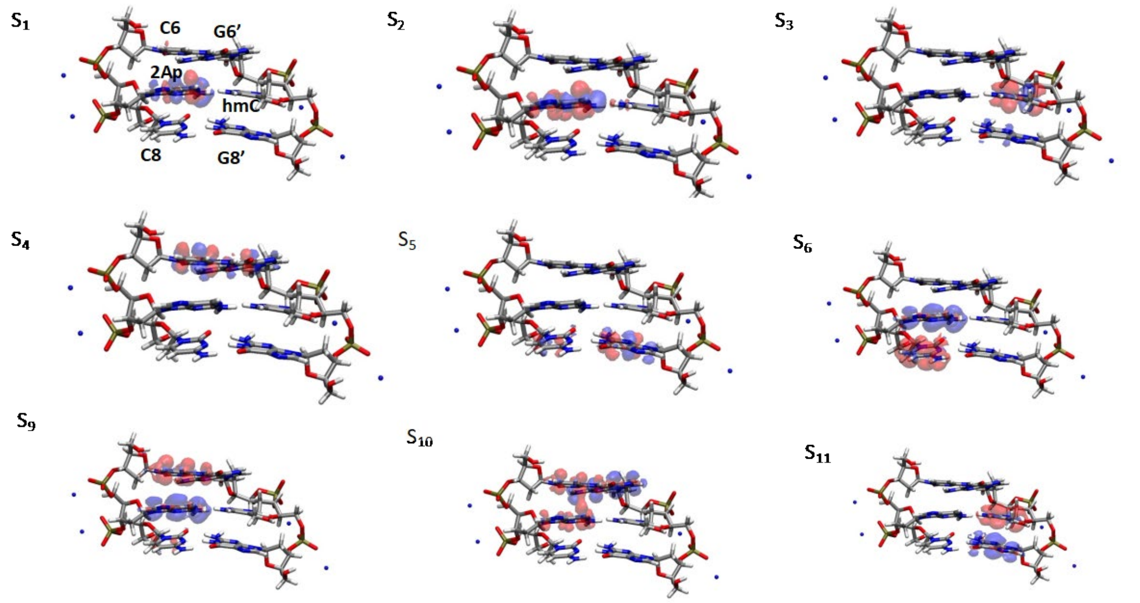

Figure S8. Excited-ground state density difference for hmC-stacked system. Blue means from where the electron moves to whereas red means where the electron goes to.

Table S6. S_n (n refers to the optimized Franck-Condon state), excited state character of different S_{1min}, vertical emission (VEE) and adiabatic energies (AE, respect to the ground state at Franck-Condon) in eV, oscillator strength and charge transfer (CT) character in a.u.

S_n	Character	VEE (AE)	f	CT	S_n	Character	ΔE	f	CT
T-less stacked					T-stacked				
S_1, S_{10}	ππ* 2aP (2amP-C6)	3.78 (3.99)	0.1899	0.1	S_1, S_6	ππ* 2aP (2amP-C6)	3.80 (3.96)	0.2007	0.1
S_8	CT G8->T	3.18 (3.96)	0.0058	0.6	S_9	CT G8->T	3.30 (4.09)	0.0087	0.8
hmC-less-stacked					hmC-stacked				
S_1	ππ* 2aP	3.96 (3.60)	0.2952	0.0	S_1, S_6	ππ* 2aP (2amP-C6)	3.70 (4.07)	0.1562	0.2
S_8	CT G8->hmC	3.34 (3.63)	0.0292	0.6	S_{11}	Dimer GhmC	CI		
S_9	CT 2aP->C8	3.43 (3.62)	0.0255	0.7					

Table S7. Fluorescence decay profile of matched and mismatched 2Ap-labeled dsDNA sequences. At 290 nm excitation and 370 nm emission.

**Sequences**	τ_1 **(ns)**	α_1	τ_2 **(ns)**	α_2	τ_3 **(ns)**	α_3	τ_{av} **(ns)**	$\chi 2$
C2ApC	0.59	0.91	3.05	0.07	9.99	0.02	0.95	1.1
C2ApC/GTG	0.70	0.69	2.72	0.23	10.84	0.08	1.98	1
C2ApC/GCG	0.64	0.73	2.58	0.2	10.40	0.07	1.68	1
C2ApC/GmCsG	0.69	0.73	2.89	0.2	10.80	0.07	1.83	1.1
C2ApC/GhmCsG	0.68	0.74	2.77	0.19	10.60	0.07	1.75	1

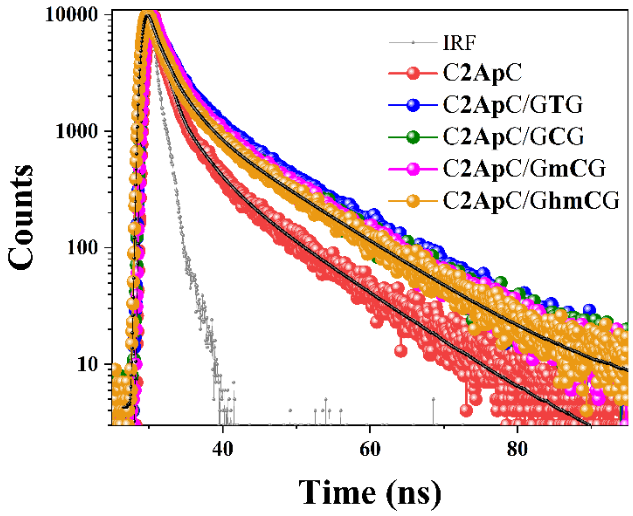

Figure S9. The fluorescence decay of both matched and mismatched DNA duplexes is shown at a maximum emission wavelength of 370 nm.

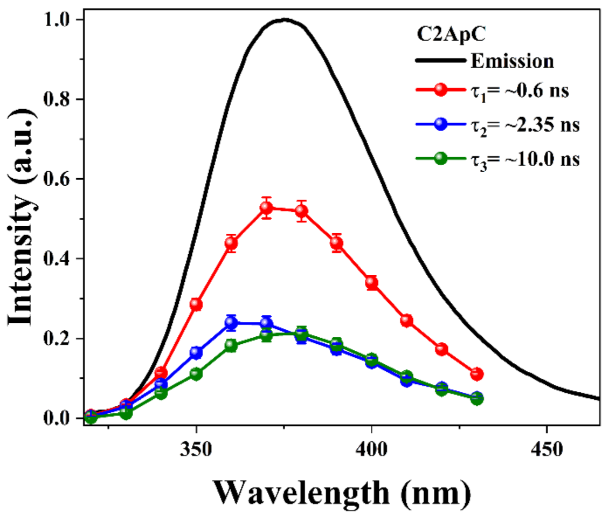

Figure S10. DAS spectra of 2Ap-labeled ssDNA sequence. The black lines denote the steady-state fluorescence spectra.
